# Activity-based CO_2_ sensing using CarboSenR2 provides new insights into cellular metabolism

**DOI:** 10.1016/j.redox.2026.104067

**Published:** 2026-02-04

**Authors:** Ben Reddan, Rawan Shahen, Rafael Radi, Mia McCalmont, Ori Green, Eoin P. Cummins

**Affiliations:** a School of Medicine, University College Dublin, Dublin, 4, Ireland; b Conway Institute of Biomolecular and Biomedical Science, Dublin, 4, Ireland; c Schulich Faculty of Chemistry & the Resnick Sustainability Center for Catalysis, Technion, Israel Institute of Technology, Haifa, 32000, Israel; d Departamento de Bioquímica, Facultad de Medicina, Universidad de la República, Montevideo, Uruguay; e Centro de Investigaciones Biomédicas (CEINBIO), Facultad de Medicina, Universidad de la República, Montevideo, Uruguay

**Keywords:** Carbon dioxide, CO_2_, Sensor, Mitochondria, Metabolism, CO_2_ cell-imaging

## Abstract

Carbon dioxide (CO_2_) is an ancient and ubiquitous physiological gas that is produced during aerobic respiration, consumed during photosynthesis and is present in the Earth's atmosphere at steadily increasing levels in modern history. CO_2_ has often been considered a simple waste product of metabolism and has to date garnered considerably less research activity compared to that of oxygen, the substrate of aerobic respiration. However, recent research has demonstrated important roles of CO_2_ in immunometabolism, immunology, skeletal and smooth muscle physiology, epithelial cell behaviour, cellular signalling and clinical medicine. Identification of CO_2_ dependent post-translational modifications using recently developed mass spectrometric approaches has directly linked CO_2_ to protein function (independent of CO_2_ -associated changes in pH) strengthening the argument for further research in this area. Notably, there has been a lack of reliable tools to directly monitor CO_2_ in living systems to date. CarboSenR2 is a new CO_2_ selective fluorescent molecular sensor which has not been fully evaluated *in vitro* and has not been specifically applied to study CO_2_ production *in cellula*. Here, we demonstrate the utility of CarboSenR2 as an activity-based CO_2_ sensor in multiple cell systems using flow cytometric and microscopy based approaches. These data demonstrate that CarboSenR2 is sensitive to CO_2_ concentrations within the physiological and pathophysiological range observed in humans and reveal the intriguing presence of mitochondrial-associated R-Dye microdomains within cells. Thus, these findings highlight the potential of CarboSenR2 to facilitate new investigations into the role and dynamics of CO_2_ in physiological systems.

## Graphical abstract

**Figure:**
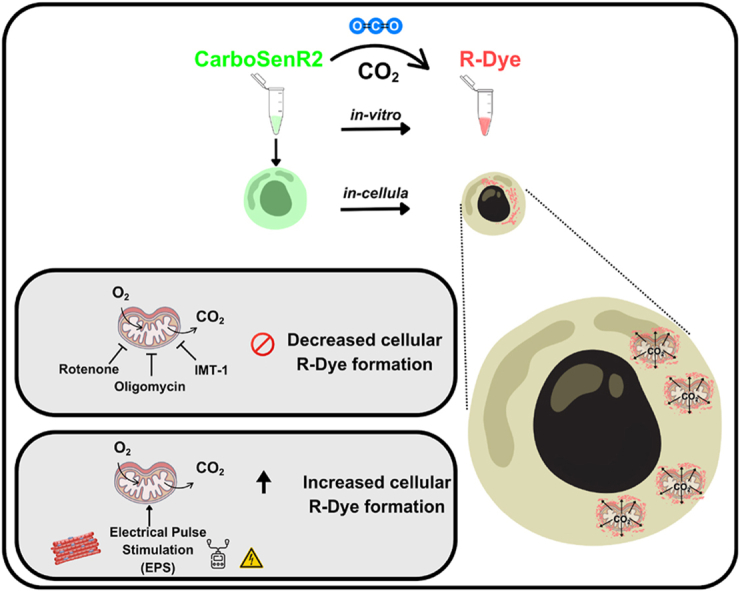

## 1 Introduction

**Fig. 1:**
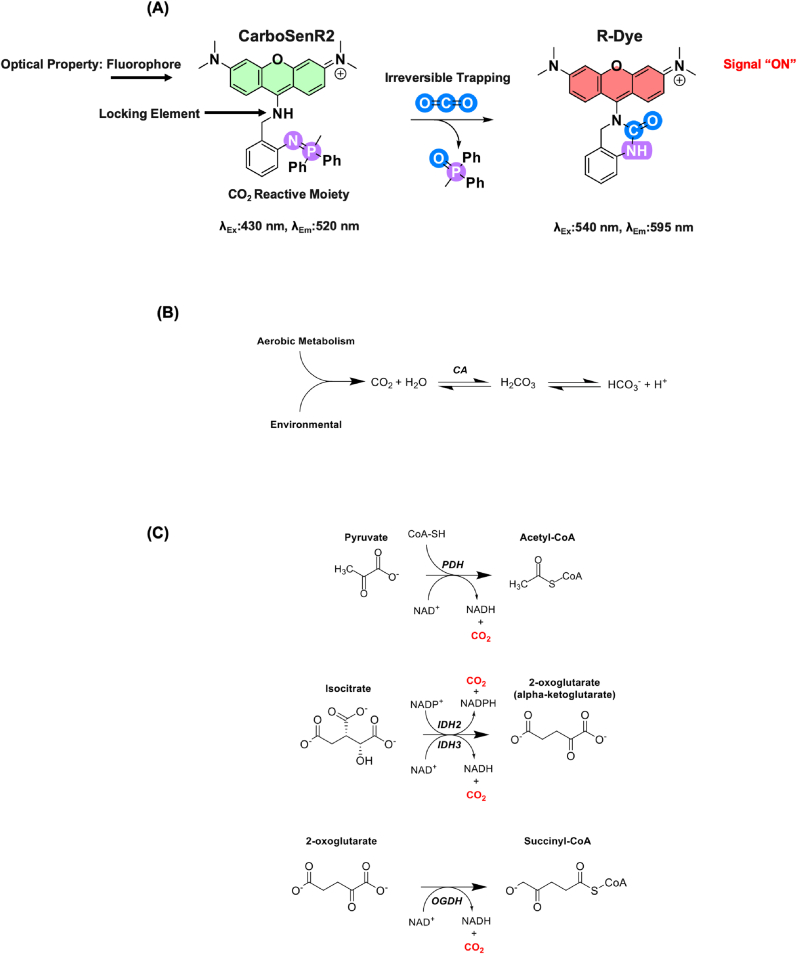
**Activity-Based Sensing of CO_2_ Using CarboSenR2 and the CO_2_–Bicarbonate Equilibrium in Mammalian Tissues. (A)** Schematic representation of CarboSenR2's mechanism for CO_2_ detection, highlighting its cleavage and bathochromic shift upon CO_2_ interaction. **(B)** Exposure to metabolic or environmental CO_2_ leads to hydration into carbonic acid (H_2_CO_3_), and equilibrium with bicarbonate (HCO_3_^−^), which is mediated by carbonic anhydrase (CA). **(C)** Mitochondrial enzyme reactions during aerobic respiration serve as major intracellular sources of CO_2_. Key contributors include the Pyruvate Dehydrogenase Complex (PDH), Isocitrate Dehydrogenase 2/3 (IDH2/3), and Oxoglutarate Dehydrogenase (ODGH).

Carbon dioxide is a ubiquitous physiological gas, which every cell in the body is exposed to. CO_2_ is primarily produced by key enzymes in the pre-Krebs (Pyruvate dehydrogenase (PDH)) and Krebs cycle (Isocitrate dehydrogenase (IDH) and 2-oxoglutarate dehydrogenase (OGDH)) pathway during aerobic metabolism (Fig. 1C) [1,2]. While the importance of oxygen in physiological systems is widely appreciated, much less is known about the role of CO_2_ in cellular function [3]. Part of reason for the disparity in knowledge between O_2_ and CO_2_ is a historic lack of suitable and selective molecular tools to measure CO_2_ concentrations in cellular systems [4]. Indeed, much of the data in the scientific literature relating to physiological levels of CO_2_ is based on systemic measurements of arterial or venous blood gas, which is likely very far removed from tissue or cellular levels of CO_2_ (which is clearly the case for O_2_ [5,6]). Recently an activity-based sensing (ABS) approach was developed for the detection of molecular CO_2_ based on the principle that a unique spectral signal is produced with high selectivity in response to the target analyte (in this case CO_2_) [7]. This ABS approach led to the design and synthesis of a family of new CO_2_-activity based sensors, based on a cascade aza-Wittig reaction (the ability of iminophosphoranes to react with CO_2_ under mild conditions). In this work, we utilized CarboSenR2, a CO_2_-responsive fluorescent sensor. It consists of a pyronine-based fluorophore linked to a CO_2_ responsive phosphazene-containing amine linker. In the absence of CO_2_, the sensor exhibits green fluorescence (λEx = 430 nm, λEm = 520 nm, Fig. 1A). Upon exposure to CO_2_, the phosphazene undergoes an aza-Wittig reaction to form a transient isocyanate intermediate, which subsequently reacts with the pendant amine to generate a stable urea-based fluorophore named R-Dye with distinct optical properties (λEx = 540 nm, λEm = 595 nm Fig. 1A). In physiological systems, CO_2_ can act as a vital signalling molecule across all domains of life - by regulating blood pH, cellular respiration & proinflammatory signalling in mammals [[8], [9], [10]], serving as a key substrate for photosynthesis in plants [11], and influencing beta-lactamase activity in bacteria [12]. Additionally CO_2_ has been recently recognised to modulate peroxide metabolism and signalling in biological systems [2,13,14]. It is evident that studying the function of CO_2_ in biological systems deepens our understanding of its role in mammalian physiology and disease. The ability of CarboSenR2 to respond to CO_2_ exposure has significant potential to be used to study CO_2_ in *cellula*. While this ABS sensing approach has been demonstrated *in vitro* using exogenous CO_2_ [7], its utility in sensing physiological changes in CO_2_ concentrations has not yet been specifically performed *in cellula*. In order for tools such as CarboSenR2 to be of value in cellular and redox biology they must be reliably taken up by different cells at low concentrations (to minimise cytotoxic effects) and function within the physiological to pathophysiological range seen in humans. Here we characterise the use of CarboSenR2 using flow cytometric and imaging approaches to demonstrate its utility as an activity-based sensor in biological systems. In the broader context, these results validate the underlying activity-based sensing strategy, confirming its suitability for biological imaging and gives opportunities for future developments.

## 2 Materials & methods

### 2.1 Cell culture

#### 2.1.1 THP-1 monocytes

THP-1 monocytes were maintained at a density of 2 × 10^5^-1x10^6^ cells/ml and cultured twice per week, with passage number continuously tracked. All experiments were carried out below P30 to prevent unwanted effects due to aging. All procedures and treatments prior to cell lysis were performed in a Class II biological safety cabinet or a CO_2_ chamber, as described below. For cell culture, cells were counted and media containing the appropriate number of cells was transferred to a T75 tissue culture flask containing fresh media, to a total of 20-30 mls. THP-1 cells were maintained in RPMI 1640 medium (61870036, Gibco), supplemented with 10% Foetal Bovine Serum (FBS) (10270-106, Gibco) and 1% Penicillin-Streptomycin (PenStrep) (15070-063, Gibco). Cells were maintained in incubators (Thermo Scientific) at 37 °C in 21% O_2_ and 5% CO_2_.

#### 2.1.2 C2C12 murine myoblasts

C2C12 murine myoblasts were split every 3-4 days when a 60% confluency was achieved to avoid myotube differentiation. Cells were grown in Dulbecco's Modified Eagle Medium (DMEM; 1X Gibco#11995-073) containing 4.5 g/L d-Glucose with l-Glutamine supplemented with 10% Fetal Bovine Serum (FBS; Gibco #10270-106) and 1% penicillin-streptomycin (Pen-Strep; Gibco #15070-063). Cells were maintained in incubators (Thermo Scientific) at 37 °C in 21% O_2_ and 5% CO_2_.

### 2.2 Carbon dioxide exposures

**Fig. 2:**
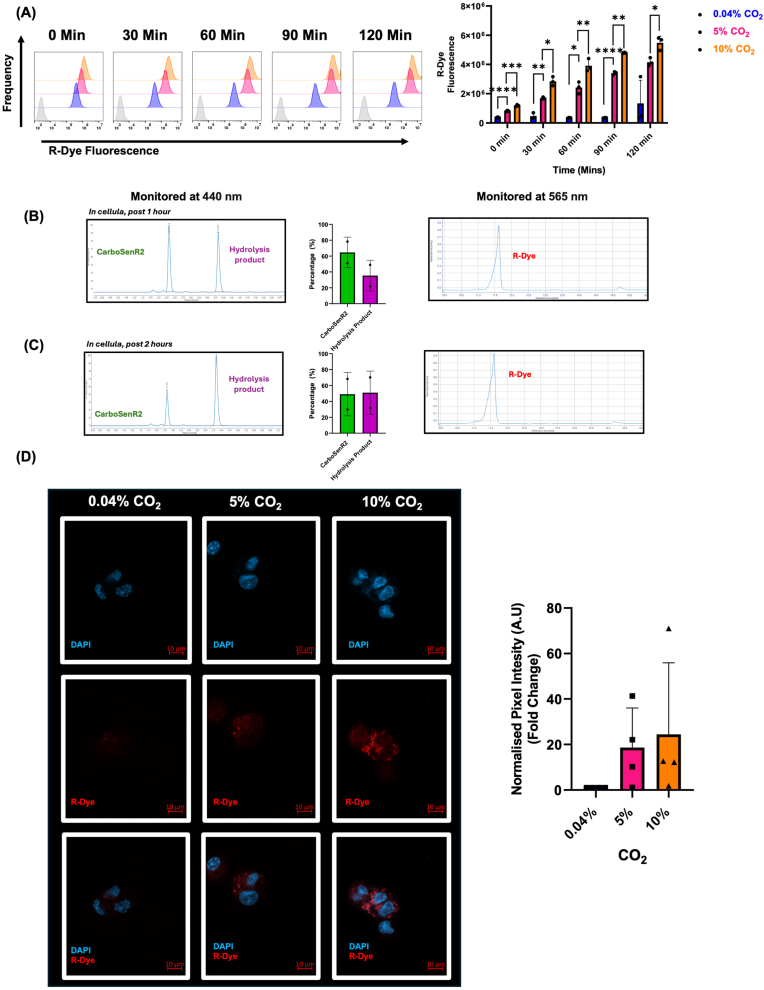
**Time & CO_2_ Response Model of CarboSenR2-Loaded THP-1 Monocytes by Flow Cytometry**. **(A)** Quantification of the time-dependent kinetics of CarboSenR2-loaded THP-1 monocytes compared to the DMSO vehicle. CarboSenR2 dye (1.5 μM) fluorescence was detected in the FITC channel (520 ± 20 nm) to confirm loading. Cells were exposed to 0.04%, 5%, or 10% CO_2_, and R-Dye fluorescence was detected in the PE channel (575 ± 20 nm). Flow cytometry was conducted using a Beckman Coulter CytoFlex LX. Data represent three independent experiments (N = 3) and are presented as geometric mean ± standard deviation. Statistical analysis was conducted using repeated measures two-way ANOVA, followed by Tukey's post-hoc multiple comparisons test. Statistical significance is indicated as follows: ∗p < 0.05, ∗∗p < 0.01, ∗∗∗p < 0.001, ∗∗∗∗p < 0.0001. LC-DAD-MS analysis of C2C12 myoblast cells loading with CarboSenR2 (5 μM) following exposure to 5% CO_2_ for **(B)** 1 h & **(C)** 2 h. CarboSenR2, hydrolysis products & R-Dye analysis using liquid chromatography monitoring absorbance at 440 nm (CarboSenR2, hydrolysis products) & 565 nm (R-Dye). **(D)** Confocal microscopy using Zeiss LSM 800 was used to analyse CarboSenR2-loaded THP-1 monocytes following 1-h exposure to 0.04%, 5%, or 10% CO_2_. Nuclei were counterstained with DAPI (*λ*_Em_ 440 nm), and R-Dye fluorescence was visualised at *λ*_Em_ 590 nm using a 63x oil immersion lens. Scale bars are indicated in all panels and represent the image scale (10 μm). Semi-quantitative analysis of pixel intensity was conducted using ImageJ analysis software. Individual data points represent that mean of three fields of view from three independent experiments (N = 4) and are presented as mean ± standard deviation.

All CO_2_ exposures were carried out in humidified environmental chambers (Coy Laboratories) maintained at 37 °C. For atmospheric experiments (Fig. 2 and Supplemental Fig. 4), CO_2_ concentrations were set at 0.04% (ambient conc.), 5% (physiological conc.), or 10% (pathophysiological conc.). In all other CarboSenR2 experiments, samples were consistently maintained at a standard background level of 5% CO_2_ at 37 °C to normalise the contribution of environmental CO_2_ to R-Dye development. Under these standardised and equilibrated background CO_2_ conditions, any changes in R-Dye fluorescence are interpreted to be a consequence of cellular metabolic contributions to intracellular CO_2_. Experiments utilized atmosphere specific buffered, phenol red-free DMEM (D1152, Sigma Aldrich) (4500 mg/L glucose, l-glutamine and 25 mM HEPES, 10% FBS Gibco #10270-106, 1% Penicillin-Streptomycin (PenStrep) (15070-063, Gibco) supplemented with: 0 g NaHCO_3_/500 ml, 2.21g NaCl/500 ml **0.04% CO_2_ media**, 1.21 g NaHCO_3_/500 ml, 0.78g NaCl/500 ml **5% CO_2_ media,** or 2.21 g NaHCO_3_/500 ml, 0g NaCl/500 ml **10% CO_2_ media**. Media was pre-equilibrated to the different CO_2_ environments in advance of experiments to ensure medium CO_2_/NaHCO_3_^−^ equilibrium prior to the initiation of experiments (Supplemental Table 1). This model previously demonstrated that extracellular and intracellular pH remains stable in THP-1 cells cultured under these pH- buffered conditions [15,16]. For all *in cellula* experiments, cells were seeded at the density indicated in the respective protocols below.

### 2.3 Nonyl acridine orange staining

Acridine orange 10-nonyl bromide (A1372, Merck) was applied to assess mitochondrial cardiolipin content [17]. Cardiolipin is a phospholipid specifically present on the mitochondrial membrane which strongly correlates with mitochondrial mass. NAO emission can be detected at 519 nm. In brief, THP-1 monocyte cells were seeded on flat-bottomed 6-well plates in 2 ml phenol-free RPMI-1640 GlutaMAX media. Subsequently, a final concentration of 500 nM of NAO/PBS was prepared and added to each well in a 6-well plate at a ratio of 1:2 (NAO/PBS: Media). Cells were incubated at 37 °C and 5% CO_2_ for 30 min protected from light. Cells were then washed to remove excess dye with 100 μl of PBS and prepared for flow cytometry by resuspending in 100 μL PBS. Analysis was conducted on a CytoFLEX LX flow cytometer (Beckman Coulter Life Sciences). Background was removed by subtracting negative control (cells without NAO dye) value from all samples.

### 2.4 Biotracker 405 staining

Biotracker 405 (SCT135, Sigma-Aldrich) is a fluorogenic dye used for staining mitochondria. The dye is membrane permeable and accumulates within mitochondria becoming brightly fluorescent. BioTracker 405 is specifically designed for assessing mitochondrial membrane potential (ΔΨm), a critical indicator of mitochondrial function. The chemical structure of BioTracker405 is not disclosed by the manufacturer. Biotracker 405 emission can be detected at 440 nm. In brief, THP-1 monocyte and C2C12 myoblast cells were seeded in respective 35 mm plates in 2 ml of phenol-free RPMI-1640 GlutaMAX media and incubated for 24 h. Subsequently, a final concentration of 100 nM (flow cytometry) or 400 nM (microscopy) of Biotracker 405 was spiked into the media. Cells were incubated at 37 °C and 5% CO_2_ for 30 min protected from light. Cells were then prepared and analysed by either flow cytometry or microscopy.

### 2.5 LysoTracker Deep Red staining

LysoTracker Deep Red (L12492, Invitrogen) is a cell-permeable, non-fixable, red fluorescent dye that stains acidic compartments within a cell, such as lysosomes [18,19]. The chemical structure of LysoTracker™ Deep Red is not disclosed by the manufacturer. Manufacturer documentation demonstrates its colocalization with CellLight™ Lysosome-GFP (C10596, Invitrogen), a BacMam-based lysosomal marker, suggestive of its selective accumulation within lysosomal compartments. LysoTracker Deep Red emission can be detected at 668 nm. In brief, approximately 1 × 10^5^ C2C12 myoblast cells were seeded on 35 mm glass bottom culture dishes in 2 ml of phenol-free RPMI-1640 GlutaMAX media. Cells were incubated for 24 h. Subsequently, cells were stained with LysoTracker Deep Red by pipetting a 1 mM stock solution directly into culture media at a final concentration of 200 nM and following the manufacturer's protocol. Cells were then prepared and analysed by live-cell microscopy as per methods.

### 2.6 Western blot analysis

Whole-cell protein lysates were prepared using whole cell lysis buffer (150 mM NaCl, 25 mM Tris pH 8, 1 mM ethylenediaminetetraacetic acid, 1% Triton X-100). Cytosolic protein lysates were prepared using a hypotonic lysis buffer method. Cells were first incubated on ice for 10 min in cytosolic buffer (10 mM HEPES, pH 8; 1.5 mM MgCl_2_; 10 mM KCl; 200 mM sucrose; 0.5 mM DTT; 0.25% NP-40 (IPEGAL); protease inhibitor cocktail (PIC) 1:100), then scraped and centrifuged. The resulting pellet was resuspended in nuclear lysis buffer (20 mM HEPES, pH 8; 420 mM NaCl; 0.2 mM EDTA; 1.5 mM MgCl_2_; 0.5 mM DTT; 25% glycerol; PIC 1:100) and incubated on ice for 30 min, followed by centrifugation. The supernatant, representing the nuclear protein fraction, was collected. All lysis buffers were supplemented with protease inhibitor cocktail (P2714, Merck).

Lysates were quantified using the DC Protein Assay kit (Biorad, Hercules, CA, USA) before sodium dodecyl sulfate–polyacrylamide gel electrophoresis on the BioRad mini-protean system. Wet transfer was performed onto nitrocellulose membranes and reversibly stained with Revert 700 total protein stain (LI-COR, Lincoln, NE, USA). Membranes were washed in Revert 700 wash solution and imaged at 700 nm (LI-COR), then destained in Revert 700 destaining solution (Li-Cor) prior to immunoblotting. Membranes were blocked in 5% milk in TBST for 1 h followed by overnight incubation in primary antibody (1:1000) at 4 °C (phospho Acetyl-CoA Carboxylase (Ser79) (CST-3661S, Cell Signalling), PGC-1a (A12348, ABclonal), Lamin A/C (CST-4777S, Cell Signalling), mitochondrial cytochrome *c* oxidase subunit 1 (ab14705, Abcam)). Fluorescent secondary goat anti-mouse or anti-rabbit antibody (IgG (H + L) Goat anti-Rabbit, DyLight 800 4X PEG,: 10733944, Fisher Scientific, IgG (H + L) Cross-Adsorbed Goat anti-Mouse, DyLight® 680,: 10797775, Fisher Scientific) incubations were performed at a 1:2000 dilution and imaged at 680 nm or 800 nm. Membranes were imaged using a LiCor Odyssey CLx imager and analysed using Empiria Studio image analysis software (LiCor, version 2.3.0.154). Nuclear fractions were immunoblotted for the protein of interest and Lamin A/C, with protein expression levels normalised to Lamin A/C as a loading control for nuclear protein content.

### 2.7 Cell counting & viability assessments

A 10 μL aliquot of cells was drawn from each well and mixed 1:1 with 10 μL of Trypan Blue (15250-061, Gibco) solution. The mixture was thoroughly mixed, and 10 μL was loaded onto a haemocytometer. Cells were counted in three fields, and the average count was calculated. To account for the 1:1 dilution with trypan blue, total cell numbers were multiplied by two. Viable cells were identified as clear (unstained) due to intact membranes, while non-viable cells appeared dark blue, indicating loss of membrane integrity.

#### 2.7.1 MTT assay

The MTT reductase assay [3-(4,5-dimethylthiazol-2-yl)-2,5-diphenyltetrazolium bromide] measures the reduction of MTT to insoluble formazan by intracellular reductase enzymes. This conversion occurs predominantly within mitochondria, but also in other intracellular organelles. Although the MTT assay is commonly used as a proxy for cellular viability—given that intracellular reductase activity often correlates with viable cell number—its principal readout reflects cellular reductase activity, which is closely linked to enzymatic and mitochondrial function. Briefly, approximately 1 × 10^6^ THP-1 monocytes per well were loaded with CarboSenR2 (1.5 μM) and seeded in 150 μL of 5% CO_2_-buffered DMEM in flat-bottom 96-well plates. MTT stock solution (5 mg mL^−1^; Sigma-Aldrich, M2128-1g) was also added at a media-to-dye ratio of 3:1, followed by incubation for 1 h at 37 °C in a 5% CO_2_ atmosphere, protected from light. Cells were subsequently centrifuged at 1500×*g* for 5 min, and the supernatant was carefully aspirated. Formazan crystals were solubilised by adding 200 μL of dimethyl sulfoxide (DMSO; Sigma-Aldrich, 472301) to each well, followed by incubation at 37 °C and 5% CO_2_ for 30 min, protected from light. Absorbance was measured at 570 nm with reference at 690 nm using a CLARIOstar plate reader (BMG Labtech). Background absorbance was corrected by subtracting values from negative control wells containing cells without MTT dye. All absorbance measurements were analysed in duplicate.

### 2.8 CarboSenR2

#### 2.8.1 CarboSenR2 constitution

CarboSenR2 stocks were synthesised by the Green group at the Schulich Faculty of Chemistry, Technion – Israel Institute of Technology. 10 mM stocks of rhodamine derivative and 10 mM stocks of diphenylmethyphosphine were kept frozen at −20 °C. Upon freeze thawing, both components were combined at a 1:1 ratio in a fume hood, protected from light, sealed from atmospheric air and left to stand for 10 min at room temperature. A 1.5 μM working solution of CarboSenR2/PBS was made and protected from light.

#### 2.8.2 Cell loading

Media was removed from cells. CarboSenR2/PBS solution was incubated with cells at a quantity of 1 ml of solution per 1 × 10^6^ viable cells. Cells were incubated for 30 min at 37 °C in an ambient CO_2_ environment. Samples were inverted every 10 min to ensure optimal loading. After 30 min, cells were removed from the incubator, centrifuged at 100xG for 5 min and wash thrice in PBS. Cells were resuspended in appropriate media and interventions performed.

### 2.9 CarboSenR2 stability *in cellula*

C2C12 myoblasts (5 × 10^6^) were suspended in 10 mL DMEM and treated with CarboSenR2 (1.5 μM) for 30 min. Cells were pelleted by centrifugation, washed twice with fresh medium, resuspended in 10 mL DMEM, and incubated for an additional 1 or 2 h. At each time point, cells were collected by centrifugation, washed twice with PBS buffer, pH = 7.4, resuspended in 10 mL distilled water, snap-frozen, and lyophilized. To the dried pellets, a 1 mL solution of 1:1 acetonitrile: water was added, vortexed, filtered, and subjected to LC–DAD-MS analysis (Agilent infinity 1260 with UV-Vis detector and an MS single quad. A 20 min gradient of 10%-100% MeCN with H_2_O containing 0.1% TFA).

### 2.10 pH sensitivity of CarboSenR2 & R-dye *in vitro*

#### 2.10.1 Preparation of Britton–Robinson buffer (10 mM, pH 4–9)

Britton–Robinson (B–R) universal buffer solutions were prepared at a total concentration of 10 mM. Acetic acid (3.33 mM, 0.33 mL), phosphoric acid (3.33 mM, 0.19 mL), and boric acid (3.33 mM, 20.63 mg) were combined in a 100 mL volumetric flask and diluted with deionized water to 80 mL. The pH of the mixture was adjusted to the desired values (pH 4–9) by the dropwise addition of 1 M NaOH under continuous stirring, monitored with a calibrated pH meter (Eutech Instruments pH 700 benchtop meter).

#### 2.10.2 Fluorescence and absorbance spectral measurements over a pH range *in vitro*

Solutions of CarboSen-R2 and R-Dye (50 μM) were prepared in DMSO. For plate reader experiments, 180 μL of Britton–Robinson buffer (pH 4–9) was dispensed into each well of a 96-well microplate, followed by the addition of 20 μL of dye stock solution to give a final concentration of 5 μM (10% DMSO v/v) in a total volume of 200 μL. Absorbance and fluorescence spectra were recorded over a pH range of 4-9 using an Agilent BioTeck2 plate reader with a monochromator. All measurements were performed in triplicate, and data are presented as mean ± standard deviation.

#### 2.10.3 CarboSen-R2 end-point measurements over a pH range *in vitro*

A 50 μM stock solution of CarboSen-R2 was prepared in DMSO. For end-point assays, 2.7 mL of Britton–Robinson buffer (pH 4–9) was placed in a 4 mL vial, followed by the addition of 300 μL of CarboSen-R2 solution (final concentration: 5 μM, 10% DMSO v/v). Samples were either treated with 20 mM CO_2_ (1.5 mL) from a compressed gas source or maintained under ambient CO_2_ conditions and stirred continuously. After 2 h, fluorescence emission was recorded at *λ*_Ex_ 530 nm and *λ*_Em_ 600 nm. All measurements were performed in triplicate, and data are reported as mean ± standard deviation.

### 2.11 Confocal microscopy

#### 2.11.1 THP-1 monocytes

5 × 10^5^ THP-1 cells were seeded onto individual 18 mm coverslips (631-0153, VWR International) placed inside a 6 well plate and stimulated with 20 nM of phorbol 12-myristate 13-acetate (PMA) (16561-29-8, MP Biomedicals) for 48 h to induce cellular adhesion while preventing excessive polarisation. PMA was then removed, cells were washed with PBS and fresh media was added for 24 h. Cells were loaded with CarboSenR2 as per sensor loading protocol and exposed to ambient, 5% or 10% CO_2_ respectively for 60 min. Unstained cells were used as controls.

#### 2.11.2 Slide preparation

Cells were fixed with 4% paraformaldehyde (P6148, Sigma-Aldrich) solution in PBS for 20 min at room temperature. Cells were then washed with PBS thrice. Then, cells were permeabilised with 0.1% (v/v) Triton X-100 (648466, EMD Millipore) for 10 min at room temperature. Cells were washed thrice with PBS. Nuclei were fluorescently stained with 4,6-Diamidino-2-phenylindole, dihydrochloride (DAPI) (A1001.0010, Panreac) (300 nM in PBS) for 5 min at room temperature. Following staining, cells were washed with PBS thrice for 5 min. Coverslips with cells were mounted on 18 mm glass microscopic slides (631-0153, VWR International) with a drop of the mounting agent, Mowiol (81381, Sigma-Aldrich).The slides were allowed to cure for 3 h at room temperature while protected from light.

#### 2.11.3 Zeiss LSM 800 Airy Scan

The samples were imaged using a Zeiss LSM800 Airy Scan microscope, with the 63 × /NA 1.4 PlanApo oil lens. For excitation, diode 405, 488, and 560 nm lasers were used to excite DAPI (blue), rhodamine derivative (green) and R-Dye (red) respectively. Airy processing, image smoothening and TIFF exporting was performed with Zeiss ZENN software (Version 3.7, Carl Zeiss AG). All data were acquired with the same lasers, detectors, pixel size, and pixel dwell time parameters. For THP-1 environmental investigations, laser parameters for respective lasers are as follows: 405 nm – laser power +0.7%, pixel time 2.06 μs, detector gain 897V, digital gain 1. 488 nm – laser power +2.2%, pixel time 2.06 μs, detector gain 945V, digital gain 1, 560 nm – laser power +2%, pixel time 2.06 μs, detector gain 934V, digital gain 6. Fluorophore intensity & colocalization analysis were performed using pixel intensity quantification and overlap quantification within the ImageJ analysis software.

### 2.12 Live-cell microscopy

#### 2.12.1 C2C12 myoblasts

1 × 10^5^ C2C12 murine myoblast cells were seeded onto individual 35 mm glass-bottom culture dishes (81156, Ibidi) and cultured until a confluence of approx. 80% was achieved. Cells were not differentiated into polynucleated myotubes. Cells were loaded with CarboSenR2, Biotracker 405 or lysotracker as per loading protocol and exposed to 5% CO_2_ for 3 h. Unstained cells were used as controls.

#### 2.12.2 Zeiss Cell Discoverer 7

The samples were imaged using a Zeiss Cell Discoverer 7 microscope with full environmental control chambers allowing accurate control of temperature at 37 °C and 5% CO_2_. Images were captured using a 50x/1.2 NA water immersion objective (offering 25×/1.2 NA, 50x/1.2 NA, 100x/1.2 NA). Zeiss Cell Discoverer 7 microscope is equipped with a QBP filter (425 ± 30, 514 ± 30, 592 ± 25, 709 ± 100) and a TBP filter (467 ± 24, 555 ± 25, 687 ± 145). For excitation, diode 385, 570, 567 and 625 nm lasers were used to excite Biotracker 405 (blue), rhodamine derivative (green) R-Dye (red) and LysoTracker Deep Red (pink) respectively. Images are captured on an Axiocam 712 monochrome camera featuring a Sony CMOS chip (4096 × 3008 pixels) with a 3.45 μm x 3.45 μm pixel size. Image processing, image smoothening and TIFF exporting was performed with Zeiss ZENN software. Colocalization analysis was performed using pixel overlap quantification within the ImageJ analysis software.

### 2.13 Flow cytometry

#### 2.13.1 Sample preparation

Cells were centrifuged at 100xG and the supernatant was discarded. Cells were resuspended in 1.5 μM CarboSenR2/PBS solution (1 ml per 1 × 10^6^ cells) and incubated in a 37 °C ambient CO_2_ incubate for 30 min, inverting every 10 min, protected from light. Cells were centrifuged at 100xG and supernatant was discarded to remove excess dye. Cells were plated and intervention (indicated in figure legend) was carried out in a 5% CO_2_ chamber unless stated otherwise, protected from light. Following intervention, cell were collected in a 15 ml falcon tube and centrifuged at 100xG with supernatant disregarded. Cells were washed thrice with warm PBS. After the final wash, cells were resuspended in 100 μl of FACS buffer (5% FBS & PBS) and analysed using Beckman Coulter CytoFlex LX. Unstained controls were prepared in the same manner, with DMSO instead of CarboSenR2.

#### 2.13.2 Cytometry parameters

Upon resuspending cells in 100 μl of FACS buffer in a 1.5 ml microcentrifuge, tubes were placed in the holder of a Cytoflex S flow cytometer (Beckman Coulter). For CarboSenR2 investigations, the blue laser (488 nm) was used to excite the green rhodamine fluorophore and emission was measured in the fluorescein isothiocyanate (FITC) channel (520 nm ± 20 nm). Additionally, the R-Dye fluorophore was excited using the yellow laser (561 nm) and was measured in the phycoerythrin (PE) channel (585 nm ± 20 nm). Cells were selected for analysis based on the forward and side scatter and subsequently single cells were selected based on forward scatter area and forward scatter height. Confirmation of sensor loading in cells was verified by an increase in FITC fluorescence between sensor loaded samples and unstained control, further allowing for the quantification of cellular uptake of CarboSenR2. Changes in cellular CO_2_ production were analysed by changes in the 585 nm fluorescent signal measured within the PE channel. FlowJo™ (version 10, FlowJo LLC) flow cytometry analysis software was using to analysis flow cytometry data acquired from the experiments. Data was reported as geometric mean, adjusted for background controls. Relative fluorescence were input to Prism (version 8, GraphPad) statistical analysis software. Data analysis was conducted as stated in figure legends. Gating parameters are displayed in Supplemental Fig. 6.

### 2.14 Electrical pulse stimulation

#### 2.14.1 Differentiated myotubes

C2C12 myoblasts were cultured in 6-well plates to ∼90% confluence and differentiated with 2% horse serum containing DMEM for 6 days to form mature myotubes. Myotubes were loaded with 1.5 μM CarboSenR2 as per loading protocols and covered with 3 ml of DMEM culture media. EPS was applied using a C-Pace 100 system (C-Pace EM IonOptix) (2 ms pulse duration, 25Hz, 11.5 V) via carbon electrodes for 1 h at 37 °C under 21% O_2_ and 5% CO_2_. Flow cytometry and western blotting of myotubes was performed as described above.

#### 2.14.2 Myoblasts

Electric pulse stimulation (EPS) was performed on C2C12 cells myoblasts cultured in 6-well plates to ∼60% confluency to prevent differentiation. For CarboSenR2 and Biotracker 405 analysis, myoblasts were loaded with CarboSenR2 sensors or Biotracker 405 in parallel as per loading protocols and covered with 3 ml of DMEM culture media. EPS was applied using a C-Pace 100 system (C-Pace EM, IonOptix) (2 m/s pulse duration, 4 Hz, 20 V) via carbon electrodes for 1 h at 37 °C under 21% O_2_ and 5% CO_2_. Cells were then gently scraped for flow cytometry. For western blotting, unstained C2C12 myoblasts were exposed to EPS and lysed according to standard lysis protocol.

### 2.15 Pharmacological modulation

5 × 10^5^ THP-1 monocytes were seeded per well in a 24-well plate and loaded with CarboSenR2 or DMSO:PBS loading control following the standard protocol. Cells were then resuspended in culture media pre-treated with the indicated concentrations rotenone (R8875, Sigma-Aldrich), oligomycin (O4876, Sima-Aldrich) or acetazolamide (A6011, Sigma-Aldrich). After 1 h of incubation at 37 °C with 5% CO_2_, cells were collected, centrifuged, washed in PBS, and processed for flow cytometric analysis according to established procedures. For experiments examining the effects of acetazolamide on R-Dye formation, a DMSO control or rotenone treatment was included as a negative control.

### 2.16 Macrophage polarisation

THP-1 monocytes were seeded in 60 mm dishes and stimulated with 20 nM PMA for 48 h to promote adherence and differentiation. Following treatment, non-adherent cells were removed, cultures were washed with warm PBS, and fresh RPMI medium (10% FBS, 1% PenStrep) was added. Cells were rested for 24 h prior to polarisation. Mϕ macrophage differentiation was confirmed by increased CD14 (367115, BioLegend) expression using flow cytometry. Cells were then polarised for 48 h with either 50 ng/ml LPS (TLRL-EBLPS, InvivoGen) and 20 ng/ml IFN-γ (HZ-1301, ProteinTech) (classical ‘M1’ activation) or 20 ng/ml IL-4 (A42603, Thermo Scientific) (alternative ‘M2’ activation) Phenotypes were assessed by surface expression of CD80 (375407, BioLegend) (M1) using flow cytometry (Supplemental Fig. 5). Gating parameters for immunological studies are displayed in Supplemental Fig. 7.

### 2.17 Statistical analysis

Statistical analysis was performed using GraphPad Prism (Boston, MA, USA) (version8). ANOVA with appropriate recommended post-hoc analysis, or a two-tailed *t*-test was applied as indicated in the figure legends and with a level of significance set at P ≤ 0.05. Data is presented as mean ± standard deviation and shown in figures in histograms with error bars unless otherwise stated. For all *in cellula* experiments, where indicated, independent experiments represent independent biological replicates (cells cultured independently passaged for a minimum of 4 passages) conducted on at least two separate days.

## 3 Results

### 3.1 CarboSenR2 conversion to R-dye increases in a dose- and time-dependent manner in CO_2_ environments (0.04%, 5%, and 10% CO_2_)

CarboSenR2 is a new fluorescent sensor designed to detect molecular CO_2_ *in cellula* (Fig. 1A), however, the ability of this sensor to reliably discriminate between CO_2_ levels in the physiological range seen in mammalian systems has not been specifically tested *in cellula*. To achieve this, we developed a flow cytometric method to measure CarboSenR2 uptake and monitor it's conversion to R-Dye. The CarboSenR2 green fluorescence signal was analysed to assess THP-1 uptake efficiency. Flow cytometric analysis confirmed efficient uptake of CarboSenR2 sensor, with 99.9% of THP-1 cells exhibiting a significant increase in fluorescence in the FITC channel compared to unstained controls (Supplemental Fig. 6 D and 6E). To further investigate CarboSenR2's sensitivity to CO_2_, cells were exposed to biologically relevant CO_2_ concentrations (0.04% [ambient], 5% [physiological], and 10% [pathophysiological]) in pH-buffered media for 1 h. Flow cytometric analysis, visualised through pseudo-colour scatter plots, demonstrated that the red fluorescence (595 nm, R-Dye) increased in response to higher CO_2_ concentrations (Supplementary Fig. 6E). These results indicate that CarboSenR2 fluorescent sensor is sufficiently sensitive to detect differences in physiologically relevant CO_2_ levels within short incubation periods (1 h).

To ensure that CarboSenR2 was sufficiently stable for *in cellula* experiments we performed LC-DAD-MS analysis on cell lysates from C2C12 myoblasts loaded with CarboSenR2 and incubated at 5% CO_2_ for up to 2 h. After incubation *in cellula* CarboSenR2 and the hydrolysis product were detected and confirmed by UV-Vis and Mass spectrometric analysis (Fig. 2B, C and Supplemental Fig. 2A and B). The CarboSenR2 retention time peak was 12.32 min, while the hydrolysis product retention time peak was at 13.84 min. Importantly, after 2 h CarboSenR2 could be detected. For R-Dye, a single peak was identified at a retention time of 11.63 min (Fig. 2B and C), matching the predicted *m*/*z* of R-Dye (*m*/*z* = 413.2) (Supplemental Fig. 2). Taken together, these data indicate that (i) while CarboSenR2 is subject to hydrolysis *in cellula*, it is sufficiently stable to generate R-Dye within a 2 h experimental exposure (ii) R-Dye is generated within this time period. All subsequent *in cellula* experiments are carried out within 1-2 h of CarboSenR2 loading.

We further evaluated whether the three CO_2_ environments (0.04%, 5%, & 10% CO_2_) produce measurable changes in the R-Dye fluorescence across different time points (0-120 min). R-Dye fluorescence at 10% CO_2_ was significantly higher than that at 5% CO_2_ across all time points, with the greatest statistical differences observed at the 60- and 90-min time points. Furthermore, cells exposed to 0.04% CO_2_ did not show a significant increase in signal between 0 and 120 min. Notably, a significant difference was maintained when comparing 0.04% CO_2_ and 5% CO_2_ at all time points until 120 min. These results may suggest that CO_2_ concentrations >0.04 % is required for the detection of CarboSenR2 under these conditions (Fig. 2A). Moreover, to support these findings, THP-1 monocytes were treated with PMA (20 nM) for 24 h (to induce adhesion for microscopy). Cells were then exposed to 0.04%, 5%, or 10% CO_2_ for 60 min at 37 °C. The mean normalised pixel intensity (a measure of CarboSenR2's transition to the R-Dye) was more intense in a CO_2_ dose-dependent manner (Fig. 2D). Notably, after 1 h of exposure, cells cultured under 0.04% CO_2_ conditions exhibited the lowest mean pixel intensity compared to those exposed to 5% and 10% CO_2_, aligning with the trends observed in flow cytometric analyses.

### 3.2 R-dye fluorescence is not homogenously distributed within the cytoplasmic regions in C2C12 murine myoblasts: Live-Cell Fluorescence Microscopy

**Fig. 3:**
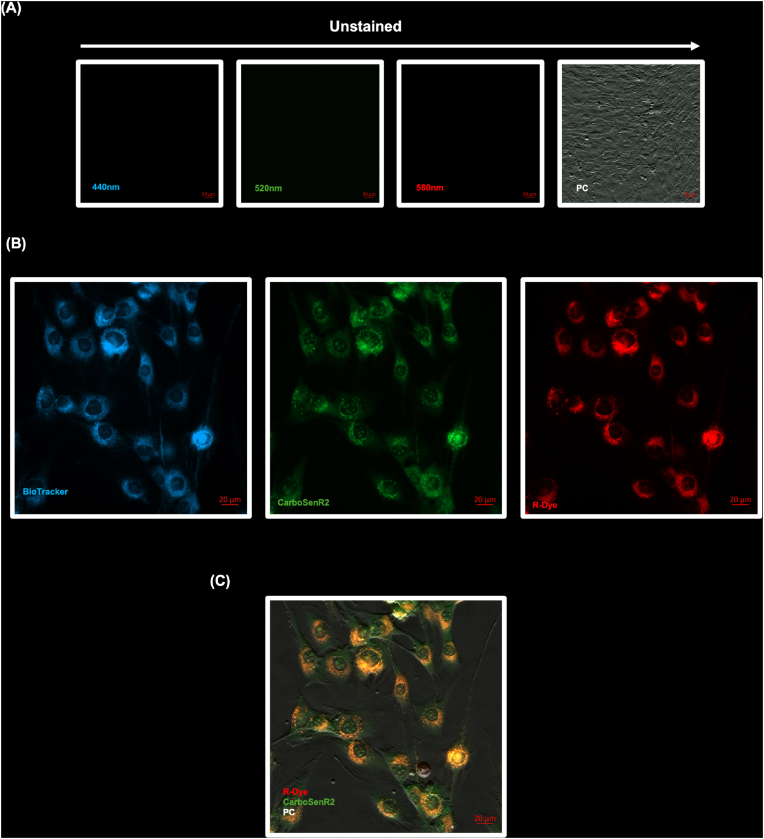
**Live-Cell Fluorescence Microscopy Reveal CarboSenR2 Dye and CarboSenR2 R-Dye display distinct sub cellular localisation in C2C12 myoblasts. (A)** Individual channels of unstained C2C12 myoblasts, with phase contrast images included to confirm cell presence. Scale bars (50 μm) are indicated in all panels **(B)** R-Dye fluorescence was captured at *λ*_Em_ 580 nm (red signal), while CarboSenR2 green fluorescence was imaged at *λ*_Em_ 520 nm. Mitochondria were counterstained with Biotracker 405 (*λ*_Em_ 440 nm). CarboSenR2 was used at a concentration of 2 μM. Live-cell microscopy was conducted using a Zeiss Cell Discoverer 7 using a 50X water immersion lens. **(C)** Overlay images showing the merged CarboSenR2 green and red signals (520 nm and 580 nm) alongside phase-contrast brightfield images of C2C12 murine myoblasts. All images are representative of three different fields of view from three independent experiments (N = 3). Scale bars (20 μm) are indicated in all panels.

The previous experiment demonstrated that CarboSenR2 is reliably taken up by cells, but gives limited information on the cellular distribution of the CarboSenR2 sensor or R-Dye. To investigate this, we loaded C2C12 myoblasts (as an adherent cell for microscopy with minimal autofluorescence) with CarboSenR2 dye and counterstained with BioTracker405 (*λ*_Em_ 440 nm) to highlight mitochondria (Fig. 3B). The cells were exposed to 5% CO_2_ for 1 h and imaged via live-cell microscopy. While the green fluorescence pattern appears relatively evenly distributed throughout the cell (predominantly in the cytoplasm but also clearly visible in the nucleus), the R-Dye fluorescence pattern is notably distinct. Interestingly, by overlaying CarboSenR2 and R-Dye fluorescence with phase contrast microscopy (Fig. 3C), we identify visible R-Dye “hotspots" indicated by the clustering of R-Dye fluorescence signals near the nuclear periphery (orange staining in Fig. 3C). Given that CarboSenR2 is relatively homogenously distributed, this suggests the presence of localised peri-nuclear, R-Dye ‘hotspots’ that appear to reside in close proximity with mitochondria.

To fully understand these results we tested whether the sensor was sensitive to pH, which could influence our interpretation of the data. CarboSenR2 is pH sensitive *in vitro* with the highest fluorescent intensities recorded at pH 4 (Supplemental Fig. 1A). This means that a high green fluorescent signal *in cellula* could indicate greater CarboSenR2 accumulation and/or localised intracellular acidosis. Interestingly, R-Dye fluorescence intensity was completely insensitive to pH in the range of 4-9 *in vitro* meaning that a high red fluorescence signal *in cellula* is not due to a localised intracellular pH environment (Supplemental Fig. 1B). Finally, *in vitro* experiments performed in the presence and absence of a CO_2_ source demonstrated that an altered pH environment is not sufficient to generate R-Dye in the absence of CO_2_ and that the rate of R-Dye generated from CarboSenR2 in the presence of CO_2_ is comparable across the physiological range (but markedly attenuated at pH 4/5) (Supplemental Fig. 1C and D).

### 3.3 R-dye signal localises in perinuclear regions and strongly colocalises with mitochondria in C2C12 murine myoblasts: Live-Cell Fluorescence Microscopy

**Fig. 4:**
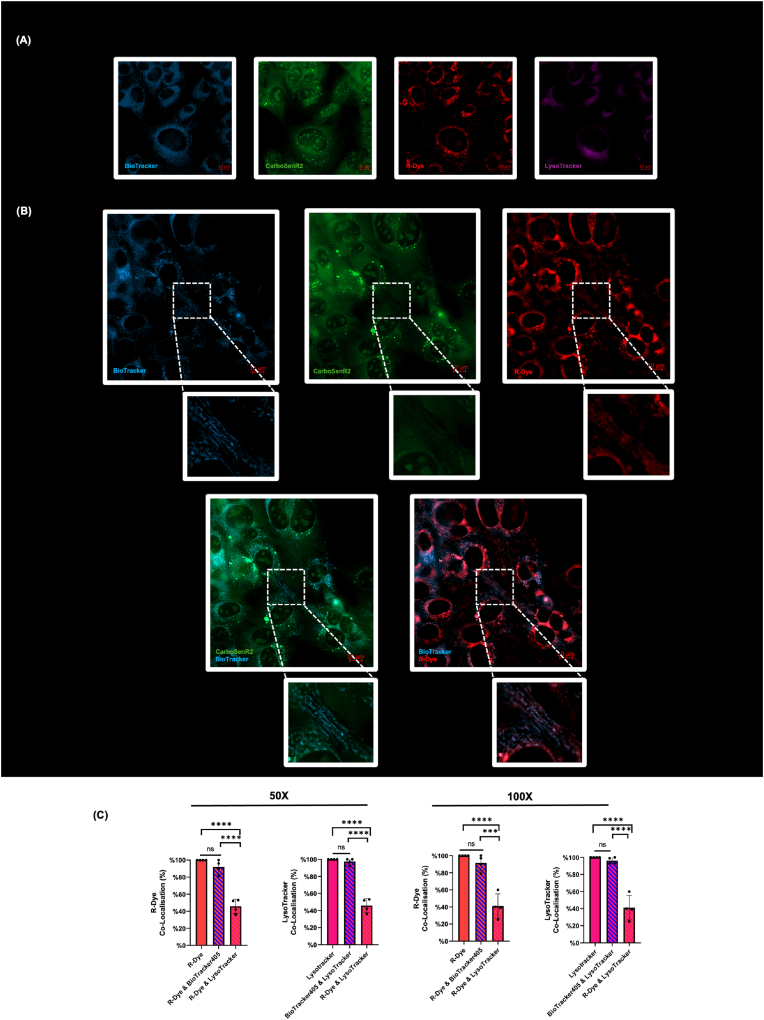
**Live-Cell Fluorescence Microscopy for Subcellular Localisation and Quantitative Analysis of CarboSenR2 in C2C12 Murine Myoblasts after 1-Hour Exposure to 5% CO_2_. (A)** Individual fluorescence channels of C2C12 myoblasts. Mitochondria were counterstained with Biotracker 405 (*λ*_Em_ 440 nm), and lysosomes were counterstained with LysoTracker (*λ*_Em_ 665 nm). CarboSenR2 fluorescence was captured at *λ*_Em_ 520 nm & R-Dye fluorescence was captured at *λ*_Em_ 580 nm. All images were captured using a 50X water immersion lens. Scale bars are indicated in all panels and represent the image scale (10 μm). CarboSenR2 was used at a concentration of 2 μM. Live-cell microscopy was conducted using a Zeiss Cell Discoverer 7. **(B)** Individual and merged fluorescence images showing CarboSenR2 fluorescence, R-Dye fluorescence, and Biotracker 405 staining using a 50X water immersion lens. Scale bars are indicated in all panels and represent the image scale (10 μm) with accompanying magnified region of interest (white box with dotted lines) depicting ‘string-like’ structures. **(C)** Quantitative analysis of the localisation of overlapping pixel areas occupied by mitochondria, CarboSenR2, and lysosomes at 50X and 100X magnification respectively. Image analysis was performed using ImageJ analysis software. Individual data points represent the mean of three fields of view from four independent experiments (N = 4) and are presented as percentage ± standard deviation relative to total R-Dye signal or lysotracker signal as indicated. Statistical analysis was performed using one-way ANOVA, followed by Tukey's post-hoc multiple comparisons test. Statistical significance is indicated as follows: ∗∗∗p < 0.001, ∗∗∗∗p < 0.0001.

To deepen our understanding of CarboSenR2's potential co-localisation with mitochondria, we conducted live-cell fluorescent microscopy in C2C12 myoblasts. Fig. 4A shows that R-Dye (red), Biotracker 405 blue (mitochondrial stain)(blue) and lysotracker (lysosome stain)(pink) co-localise in similar peri-nuclear regions imaged with a 100X water immersion lens. The Biotracker blue signal demonstrates a characteristic peri-nuclear ‘string-like’ staining of mitochondria. While the 3 distinct fluorophores do not completely overlap, they appear to be located in close proximity. In Fig. 4B we again observe a relatively homogenous distribution of CarboSenR2 (green) throughout the cell and a distinct peri-nuclear R-Dye (red) staining pattern. Focusing on the R-Dye signal we can appreciate significant overlap between the Biotracker blue signal and the R-Dye (red) signal as well as ‘string-like’ R-Dye staining (Fig. 4B and C). This suggests that R-Dye is detected within mitochondria as well as in association with mitochondria in these cells. Furthermore, quantitative analysis using ImageJ revealed significant pixel overlap between the R-Dye fluorescence (red) and Biotracker (blue), suggesting strong co-localisation between R-Dye and mitochondria (Fig. 4C). In contrast, lysotracker showed significantly less overlap with both R-Dye and Biotracker. While Biotracker and lysotracker shared some overlap, R-Dye fluorescence demonstrated <50% co-localisation with lysotracker. Taken together, these findings, imply a strong co-association between mitochondria and R-Dye fluorescence, and a much weaker association with lysosomes (another peri-nuclear cellular organelle). While CarboSenR2 is relatively homogeneously distributed (Fig. 3B,C, 4A, B), we observe distinct R-Dye fluorescence clustering in close proximity to the mitochondria (Fig. 4B & C) further suggesting that mitochondria are responsible for a significant proportion of localised CO_2_ production.

### 3.4 Rotenone, and oligomycin treatment modulate the CarboSenR2 fluorescence response in THP-1 monocytes in a dose-dependent manner

**Fig. 5:**
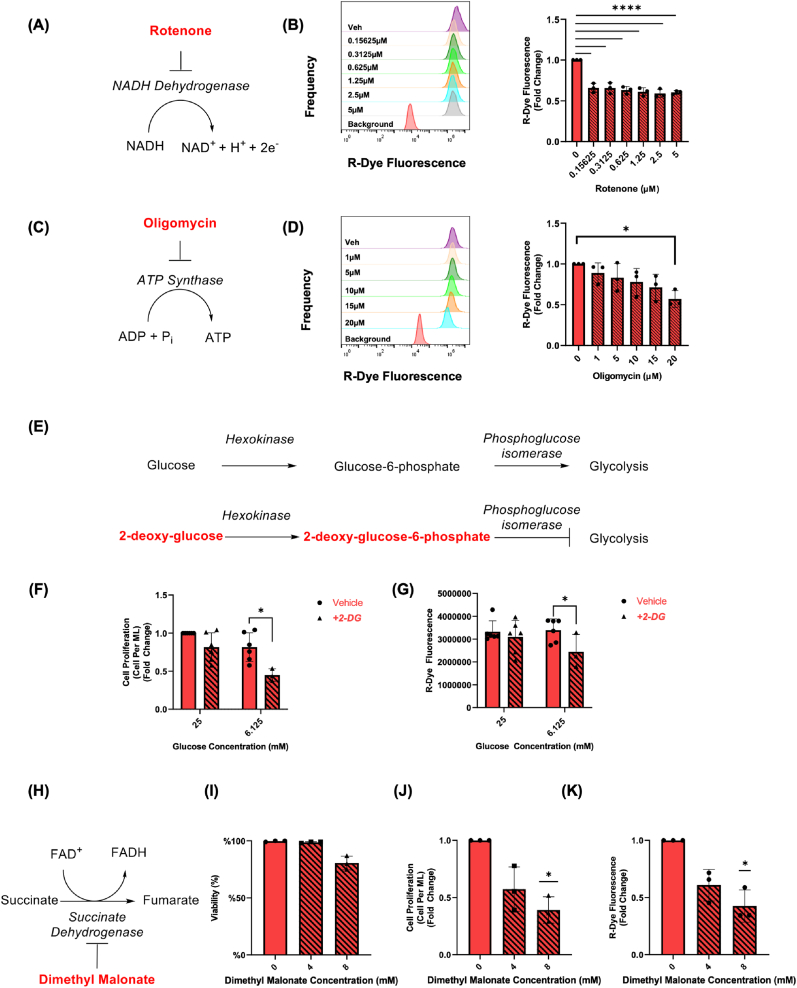
**Quantification and Correlative Analysis of CarboSenR2 Fluorescence in THP-1 Monocytes in the Presence of Metabolic Inhibitors at Various Doses (A)** Schematic representation of the mechanism of action of rotenone **(B)** Flow cytometry histograms displaying the geometric mean of PE fluorescence for CarboSenR2-loaded THP-1 monocytes, compared to the DMSO vehicle control, after treatment with varying doses of rotenone, for 1 h in a 5% CO_2_ atmosphere. CarboSenR2 was used at a concentration of 1.5 μM, and fluorescence was measured in the PE channel (575 ± 20 nm). **(C)** Schematic representation of the mechanism of action of oligomycin (**D**) Quantitative analysis of the geometric mean of R-Dye fluorescence THP-1 monocytes comparing when treated with different doses of oligomycin for 1 h in a 5% CO_2_ environment. CarboSenR2 was used at a concentration of 1.5 μM. Flow cytometry was performed using a Beckman Coulter CytoFlex LX and quantified in the PE channel (575 ± 20 nm). Data represent independent experiments (N = 3) and are presented as fold change ± standard deviation relative to vehicle control. Statistical analysis was performed using one-way ANOVA, followed by Tukey's post-hoc multiple comparisons test. **(E)** Schematic depicting the mechanism of action of 2-deoxyglucose on glycolytic enzyme reactions. **(F)** Cell proliferation rates of THP-1 monocytes treated with 25 mM of 6.125 mM glucose, with or without 2-deoxyglucose (5 mM) for 24 h. Data are presented as fold change relative to the vehicle control, with samples treated with 2-DG indicated by black striped bars. **(G)** Flow cytometry analysis of the geometric mean of PE fluorescence for CarboSenR2-loaded THP-1 monocytes, assessing changes in CO_2_ production over a 24-h period following supplementation with various doses of glucose. CarboSenR2 fluorescence was activated after 1 h of exposure in a 5% CO_2_ atmosphere, with CarboSenR2 used at a concentration of 1.5 μM. Flow cytometry was performed using a Beckman Coulter CytoFlex LX and quantified in the PE channel (575 ± 20 nm). All experiments were performed independently with N = 6, except for the samples treated with 6.125 mM glucose and 5 mM 2-DG, which were conducted independently with N = 3. Statistical analysis was conducted using two-way ANOVA, followed by Bonferroni post-hoc multiple comparisons test. Statistical significance is indicated as follows: ∗p < 0.05. **(H)** Schematic depicting the mechanism of action of dimethyl malonate on OXPHOS enzyme reactions. **(I)** Analysis of cytotoxicity using trypan blue viability assay. Data is presented as mean percentage of viable cells in THP-1 monocytes treated with malonate or vehicle control for 72 h. Data represent three independent experiments (N = 3) and are presented as fold change ± standard deviation relative to vehicle control unless otherwise stated. **(J)** Cell proliferation rates of THP-1 monocytes treated with varying doses of dimethyl malonate or vehicle control for 72 h, expressed as fold change relative to the vehicle control. **(K)** Flow cytometry analysis of R-Dye fluorescence (575 ± 20 nm) in THP-1 monocytes treated with dimethyl malonate or vehicle control for 72 h. CarboSenR2 was used at a concentration of 1.5 μM, and analysis was conducted using a Beckman Coulter CytoFlex LX, with fluorescence quantified in the PE channel (575 ± 20 nm). Statistical analysis was performed using one-way ANOVA, followed by Dunnett's post-hoc multiple comparisons test. Statistical significance is indicated as follows: ∗p < 0.05, ∗∗p < 0.01, ∗∗∗p < 0.001, ∗∗∗∗p < 0.0001. Abbreviations: 2-DG, referring to 2-deoxyglucose; 2DG-6-P, referring to 2-deoxyglucose-6-phosphate; and G6P, referring to glucose-6-phosphate.

Given our previous data indicating the existence of R-Dye ‘hot-spots’ localised to mitochondria we posited that pharmacological modulation of the mitochondrial electron transport chain for 1 h would suppress the R-Dye signal and reveal evidence of cellular CO_2_ production. Rotenone inhibits Complex I, preventing NADH oxidation, and Oligomycin inhibits Complex V, blocking ATP synthesis. Treatment of THP-1 monocytes with rotenone at concentrations ranging from nanomolar concentrations up to 5 μM, significantly decreased R-Dye fluorescence compared to the vehicle control (Fig. 5A). Treatment with oligomycin demonstrated a gradual dose dependent decrease in R-Dye mean fluorescence which becomes statistically significantly decreased when treated with 20 μM oligomycin (Fig. 5D). Addition of these compounds *in vitro* in the absence of cells did not affect R-Dye fluorescence, nor did CarboSenR2 loading affect cellular reductase activity as measured in a MTT assay (Supplemental Fig. 3 A, B, F). Taken together, these findings strongly support the concept that disruption of the mitochondrial electron transport chain at different sites with different modulators each suppress the production of R-Dye fluorescence, suggestive of suppressed endogenous cellular CO_2_ production.

### 3.5 2-Deoxyglucose and malonate supplementation decreases the CarboSenR2 fluorescence response in THP-1 monocytes

Building on the previous data we next manipulated glycolytic and mitochondrial metabolism respectively at sites distinct from those targeted in Fig. 5. 2-deoxy-d-glucose (2-DG), a competitive glucose analogue, was added to the culture media to disrupt glycolysis and inhibit glucose utilisation (Fig. 5E). Two glucose:2-DG ratios (5:1 and 1.25:1) were employed to interfere with glucose utilisation. After 24 h, cells treated with 25 mM glucose (standard RPMI culture medium glucose concentrations) and 5 mM 2-DG (5:1) showed no significant change in R-Dye fluorescence or cell proliferation. However, cells treated with 6.25 mM glucose and 5 mM 2-DG (1.25:1) exhibited a significant decrease in R-Dye fluorescence and cell proliferation (Fig. 5F and G). Additionally, we supplemented the culture media with dimethyl malonate across multiple doses (4-8 mM). While malonate itself is hydrophilic and exhibits poor membrane permeability, dimethyl malonate—a cell-permeable derivative in which the carboxylic acid groups are masked by methyl esters—can penetrate the cell membrane [20,21]. Once inside, the accumulation of intracellular malonate inhibits succinate dehydrogenase (SDH), leading to dysfunction of the electron transport chain [22], which, in theory, should alter basal CO_2_ production (Fig. 5H). Our results showed that 8 mM malonate supplementation significantly decreased R-Dye fluorescence, and significantly diminished cell proliferation (Fig. 5J and K) without a significant decrease in cellular viability (Fig. 5 I). Taken together these data support the concept that disruption of glycolysis by 2-DG and modulation of SDH activity with dimethyl malonate result in suppressed endogenous CO_2_ production.

### 3.6 Electrical pulse stimulation increases the CarboSenR2 fluorescence response in C2C12 skeletal muscle myotube cells

**Fig. 6:**
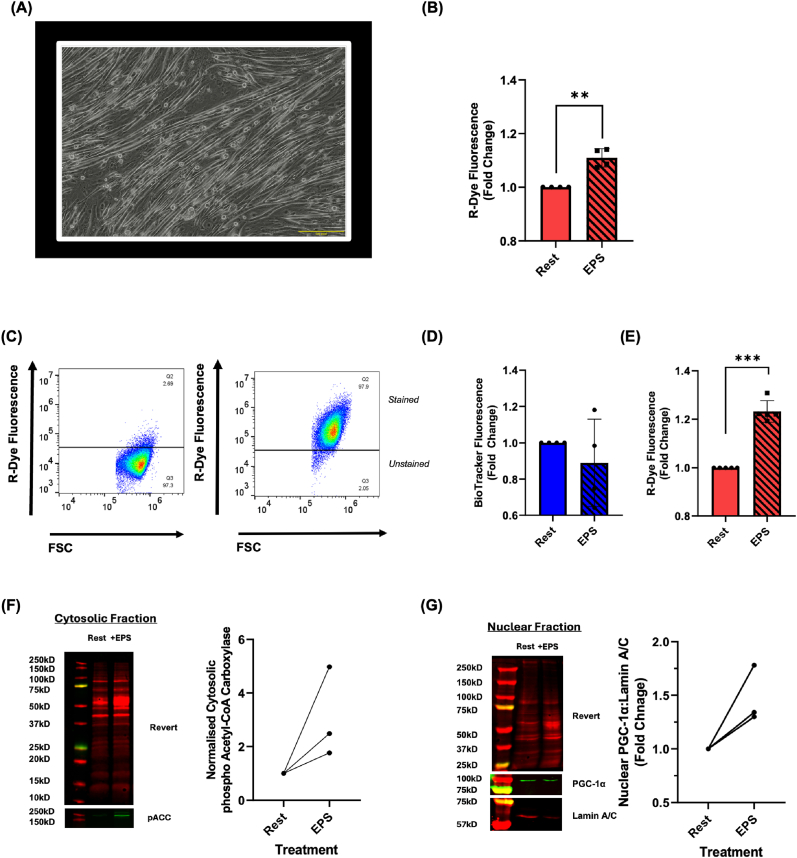
**Assessment of CO_2_ Production in CarboSenR2-Loaded C2C12 Myoblast Cells Following Exercise-Like Stimuli (A)** Visual confirmation of myoblast-to-myotube differentiation following the stated differentiation protocol. Images are representative of three fields of view & three independent experiments (N = 3). **(B)** Quantitative plots showing the geometric mean of R-Dye fluorescence (575 ± 20 nm) in C2C12 myotube cells loaded with CarboSenR2, comparing cells stimulated with EPS to those maintained at rest for 1 h in a 5% CO_2_ environment. CarboSenR2 was used at a concentration of 1.5 μM. Fluorescence was quantified using a Beckman Coulter CytoFlex LX in the PE channel (575 ± 20 nm). Data represent four independent experiments (N = 4) and are presented as fold change ± standard deviation relative to vehicle control. Statistical analysis was conducted using a paired two-tailed *t*-test. Statistical significance is indicated as follows: ∗∗p < 0.01. **(C)** Representative flow cytometry scatter plots showing the geometric mean of R-Dye fluorescence (575 ± 20 nm) in CarboSenR2-loaded C2C12 myoblast cells. **(D)** Quantitative plots showing the geometric mean of Biotracker 405 (440 ± 20 nm) in C2C12 myoblast cells comparing cells stimulated with EPS to those maintained at rest for 1 h in a 5% CO_2_ environment. Biotracker 405 was used at a concentration of 100 nM. Fluorescence was quantified using a Beckman Coulter CytoFlex LX at the stated wave length. Data represent four independent experiments (N = 4) and are presented as fold change ± standard deviation relative to vehicle control. **(E)** Quantitative plots showing the geometric mean of R-Dye fluorescence (575 ± 20 nm) in C2C12 myoblast cells loaded with CarboSenR2, comparing cells stimulated with EPS to those maintained at rest for 1 h in a 5% CO_2_ environment. CarboSenR2 was used at a concentration of 1.5 μM. Fluorescence was quantified using a Beckman Coulter CytoFlex LX in the PE channel (575 ± 20 nm). Data represent five independent experiments (N = 5) and are presented as fold change ± standard deviation relative to vehicle control. Statistical analysis was conducted using a paired two-tailed *t*-test. Statistical significance is indicated as follows: ∗∗∗p < 0.001. **(F)** Western blot analysis of phosphorylated Acetyl-CoA Carboxylase expression in cytosolic fractions of C2C12 myoblast cells following 1 h EPS stimulation. Data is expressed as fold change of protein expression normalised to total protein revert stain when comparing exercise to rest, and represents three independent experiments (N = 3). **(G)** Western blot analysis of nuclear PGC-1α expression in nuclear fractions of C2C12 myoblast cells following 1 h EPS stimulation. Data is expressed as fold change of protein expression normalised to total Lamin A/C content (nuclear loading control) when comparing exercise to rest, and represents three independent experiments (N = 3). Nuclear fractions were prepared as per methods. Lysates were probed using revert total protein stain and imaged in the 700 nm channel. Blots were incubated with a respective primary antibodies followed by a fluorescent secondary fluorophore-conjugated antibody and imaged in the 680-nm or 800-nm channel on an Li-COR imaging system. Relative intensities were quantified using Empiria Studio 3.0 Software. Image is representative of three independent experiments.

We next sought to explore CarboSenR2's potential to monitor increases in cellular CO_2_ production using an ‘exercise-like’ stimulus. Aerobic exercise leads to increased O_2_ consumption, CO_2_ production, and partially elevated extracellular lactate levels in skeletal muscle [23]. With this in mind, we stimulated mature C2C12 myotubes using electrical pulse stimulation (EPS) to mimic exercise-induced aerobic metabolism. This approach is designed to enhance CO_2_ production, stimulated by exercise-like activity. Short duration stimulations using EPS have previously been shown to induce mitophagy and upregulate PGC-1α expression [24], as well as modulate the intracellular Na^+^/K^+^ ratio and intracellular calcium influx [25]. We investigated whether 1 h of EPS affects the generation of R-Dye fluorescence, indicative of CO_2_ production in C2C12 murine myotubes. We first differentiated mature myotubes (Fig. 6A), confirmed their contraction with an EPS protocol (Supplemental Fig. 9) and observed a modest but significant increase in R-Dye in EPS-stimulated myotubes (Fig. 6B). We next investigated the effect of EPS on CarboSenR2 loaded C2C12 myoblasts. Flow cytometric gating of CarboSenR2 loaded C2C12 cells is shown in (Supplemental Fig. 6A and 6B). The C2C12 model is widely used for experiments investigating muscle atrophy, exercise, and skeletal muscle protein synthesis. Flow cytometry scatter plots again confirmed successful CarboSenR2 loading (Fig. 6C). Moreover, while EPS caused no significant changes in ΔΨm, EPS stimulation significantly increased R-Dye fluorescence when compared to resting controls (Fig. 6D and E). EPS-stimulated Acetyl-CoA Carboxylase phosphorylation as well as nuclear PGC-1α translocation indicates that the myoblasts perceived this EPS protocol, and activated pathways associated with aerobic metabolism and catabolism (Fig. 6D and E) (despite myoblasts lacking the contractile properties of mature myotubes). Taken together, these data indicate that EPS increases R-Dye production in myotubes and myoblasts indicative of increased cellular CO_2_ production.

### 3.7 THP-1-derived macrophage phenotypes exhibit distinct CarboSenR2 fluorescence profiles

**Fig. 7:**
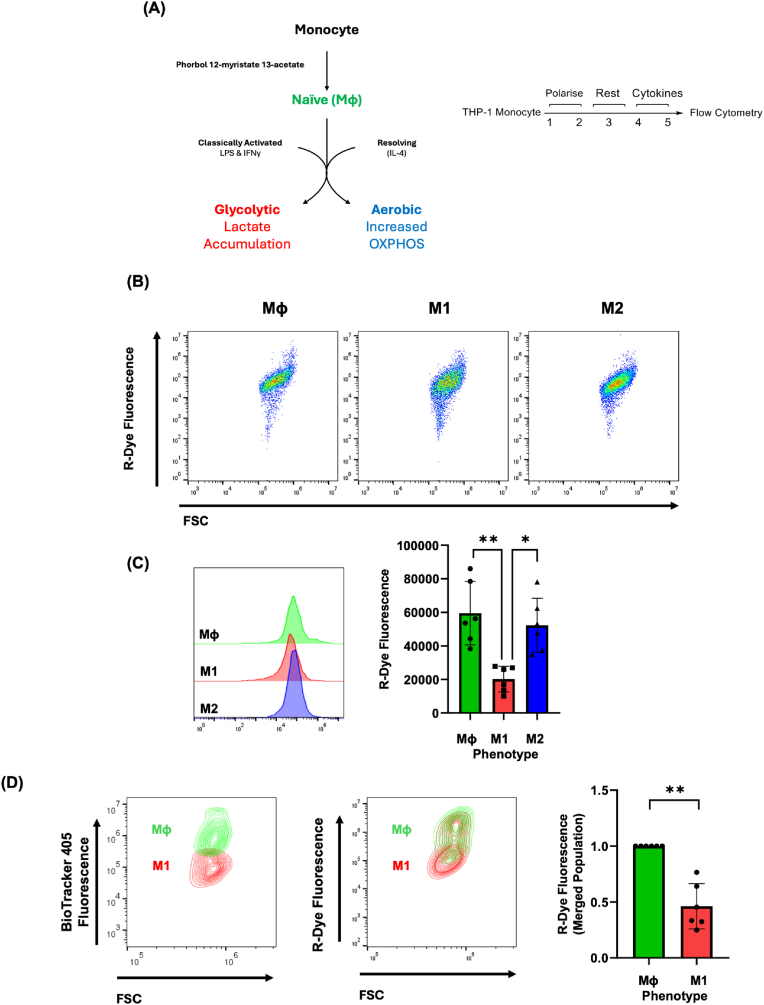
**Analysis of CarboSenR2 Fluorescence in THP-1-Derived Macrophages Following 1-Hour Exposure to 5% CO_2_ (A)** Schematic depicting the common metabolic pathways associated with each macrophage phenotype and the polarisation protocol used. **(B)** Flow cytometry scatter plots display the geometric mean of R-Dye fluorescence for CarboSenR2-loaded THP-1-derived macrophages and were quantified using a Beckman Coulter CytoFlex LX after 1-h exposure to 5% CO_2_. R-Dye fluorescence was quantified in the PE channel (575 ± 20 nm). Quantitative analysis of the geometric mean of PE fluorescence for CarboSenR2-loaded THP-1-derived macrophages **(C)** in isolation & **(D)** in co-cultures to assess CO_2_ production rates after 1-h exposure to a 5% CO_2_ atmosphere. CarboSenR2 was used at a concentration of 1.5 μM, with fluorescence quantified in the PE channel (575 ± 20 nm). Co-culture cell phenotypes identified by Biotracker 405 positive and negative signals (440 ± 20 nm). CarboSenR2 was used at a concentration of 1.5 μM. Flow cytometry was performed using a Beckman Coulter CytoFlex LX and quantified in the PE channel (575 ± 20 nm). Data represent six independent experiments (N = 6) and are shown as geometric mean ± standard deviation. Statistical analysis was performed using one-way ANOVA, followed by Tukey's post-hoc multiple comparisons test. Statistical significance is indicated as follows: ∗p < 0.05, ∗∗p < 0.01,.

Developing on our pharmacological and EPS-based experiments, we next explored whether CarboSenR2 could distinguish between macrophage phenotypes. THP-1 monocytes can be differentiated into distinct macrophage subtypes, each of which exhibit characteristic metabolic features (Fig. 7A). Classically activated (M1) macrophages predominantly engage anaerobic glycolytic metabolism, even in the presence of oxygen, leading to decreased flux through the TCA cycle and more limited mitochondrial CO_2_ production. In contrast, Mφ and alternatively activated (M2) macrophages favour oxidative metabolism, including robust TCA cycle turn over and oxidative phosphorylation [26], resulting in greater mitochondrial CO_2_ production. THP-1 monocytes were polarised into macrophages using PMA and confirmed by a significant increase of CD14 expression (Mφ) (Supplemental Fig. 7B). Polarisation to an M1-like phenotype was induced using LPS and IFN-γ and confirmed by a significant increase of CD80 expression (Supplemental Fig. 7B). IL-4 was used to induce alternative M2-like macrophage activation. THP-1 derived M1-like macrophages exhibited significantly lower levels of R-Dye fluorescence compared to Mφ and M2-like macrophages (Fig. 7C). These data suggested the potential for R-Dye fluorescence to discriminate metabolic activity between macrophages of different immune phenotypes when analysed separately. To test this further, we investigated whether R-Dye fluorescence could discriminate metabolic activity between macrophages of different immune phenotypes when analysed as a mixed cell population. To perform this we first differentiated Mφ and M1-like THP-1 macrophages as before. This time Mφ macrophages were pre-stained with BioTracker 405 (to allow us to identify the Mφ population), while M1-like macrophages remained unstained, allowing for reliable gating of each cell population (Supplemental Fig. 8). This was followed by R-Dye fluorescence quantification (in both cell types) to assess metabolic differences between the two macrophage phenotypes. Notably, the Mφ macrophage population again displayed higher R-Dye fluorescence in comparison to M1-like macrophages in this mixed cell experiment (Fig. 7D).

### 3.8 Mitochondrial polymerase inhibitor (IMT-1) decreases CarboSenR2 fluorescence response in THP-1 monocytes

**Fig. 8:**
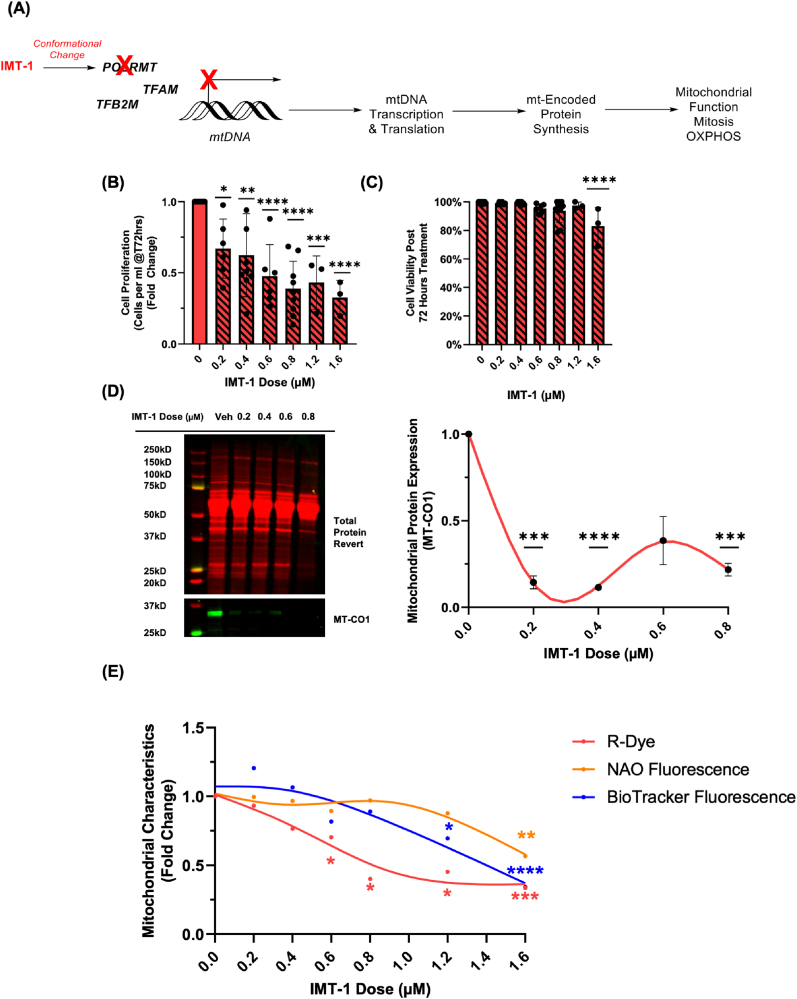
**Effects of IMT-1 on mitochondrial activity, cell proliferation, and viability in THP-1 monocytes. (A)** Schematic overview of IMT-1's inhibition of mitochondrial activity through suppression of mtDNA transcription. **(B)** Cell proliferation rates of THP-1 monocytes treated with varying doses of IMT-1 or vehicle control for 72 h, displayed as fold change relative to the vehicle control. **(C)** Trypan blue viability analysis showing the percentage of viable cells in THP-1 monocytes when treated with varying doses of IMT-1 or vehicle for 72 h. **(D)** Western blot analysis of MT-CO1 expression in THP-1 monocytes following treatment with IMT-1 or vehicle for 72 h (N = 3). Lysates were probed using revert total protein stain and imaged in the 700 nm channel. Blots were incubated with a MT-CO1 primary antibody followed by a fluorescent secondary fluorophore-conjugated antibody and imaged in the 800-nm channel on an Li-COR imaging system. Relative intensities were quantified using Empiria Studio 3.0 Software. Image is representative of three independent experiments. **(E)** Flow cytometry was conducted for the analysis of mitochondrial membrane potential (BitoTracker405, 440 ± 20 nm), cardiolipin content (Nonyl-Acridine Orange, 520 ± 20 nm), and CO_2_ (CarboSenR2, 575 ± 20 nm) in THP-1 monocytes treated with varying doses of IMT-1 or vehicle for 72 h. Data is expressed as fold change relative to the vehicle control. CarboSenR2 was used at a concentration of 1.5 μM. NAO was used at a final concentration of 500 nM. Biotracker 405 was used at a final concentration of 100 nM. Flow cytometry was conducted using a Beckman Coulter CytoFlex LX. Data represents three independent experiments (N = 3) and are presented as fold change ± standard deviation relative to vehicle control unless otherwise stated. Statistical analysis was conducted using repeated measures two-way ANOVA, followed by Tukey's post-hoc multiple comparisons test. Statistical significance is indicated as follows: ∗p < 0.05, ∗∗p < 0.01, ∗∗∗p < 0.001, ∗∗∗∗p < 0.0001.

Finally, we investigated if depleting mitochondrial protein expression (as opposed to pharmacologically modulating mitochondrial activity or metabolic intermediates) can affect the R-Dye fluorescence response. We supplemented the culture media with IMT-1 (0.2-1.6 μM), a first-in-class, specific, noncompetitive inhibitor of human mitochondrial RNA polymerase (POLRMT) (Fig. 8A). POLRMT is a key component of mitochondrial transcription, essential for OXPHOS and mitochondrial biogenesis [[27], [28], [29]]. IMT-1 disrupts mitochondrial transcription, ultimately impairing mitochondrial function and organelle integrity in both a dose- and time-dependent manner. This interference results in the mitochondria essentially becoming a hollow, protein-depleted organelle, unable to function or undergo mitosis [30]. THP-1 cells were cultured in IMT-1-supplemented media across multiple doses for 72 h. While all doses significantly decrease cellular proliferation (Fig. 8B), only the highest dose (1.6 μM) had a notable impact on cell viability (Fig. 8C). IMT-1 treatment at 0.2 μM −0.8 μM decreased MT-CO1 expression, a mitochondrial DNA-encoded complex IV protein critical for the electron transport chain function and proton transport (Fig. 8D). To evaluate the broader effects of IMT-1 on the mitochondria, we measured key markers of mitochondrial integrity and health, including mitochondrial membrane potential (BioTracker405 (*λ*_Em_ 440 nm)) and mitochondrial cardiolipin content (NAO (*λ*_Em_ 519 nm)). Concurrently, we assessed the R-Dye fluorescence response via flow cytometry following a dose-dependent treatment with IMT-1 (Fig. 8E). Notably, following IMT-1 treatment, Biotracker Blue fluorescence was significantly decreased only at 1.2 μM and 1.6 μM, while NAO fluorescence was only significantly decreased at 1.6 μM. Intriguingly, R-Dye fluorescence was significantly decreased at much lower doses of IMT-1 (0.6 μM–1.6 μM). This early and dose-dependent decrease in R-Dye fluorescence, coupled with decreased proliferation but preserved viability, suggests that fluctuations in cellular CO_2_ concentrations may serve as an early indicator of altered mitochondrial function/mitochondrial dysfunction, preceding detectable changes in mitochondrial membrane potential or mitochondrial cardiolipin content.

## 4 Discussion

Despite CO_2_'s critical role as an essential biological gas, the actual levels of CO_2_ present in biological tissues remains largely uncharacterised leading to a deficit in our understanding of the role of CO_2_ in physiology and disease. Monitoring atmospheric CO_2_, or detecting CO_2_ in solution, particularly in microscale settings such as those observed in live cells, poses significant challenges. Here we studied the ability of CarboSenR2 a novel CO_2_ fluorometric molecular sensor to detect levels of endogenous CO_2_ production using a novel combination of flow cytometric and microscopy based studies. Our results firstly demonstrate the ability of the CarboSenR2 sensor to detect differences in environmental CO_2_ concentrations which reside in the physiological to pathophysiological range (Fig. 2) over a period of 2 h. Further studies manipulating cellular metabolic function through various interventions demonstrated that the CarboSenR2 sensor is capable of detecting differences in CO_2_ steady-state concentrations. However, due to the slow reaction of CO_2_ with CarboSenR2 [7] and its competition with the fast carbonic anhydrase-catalysed hydration reaction of CO_2_ [2], the CarboSenR2-derived signal most likely reflects levels of CO_2_ once a new equilibria of CO_2_ with HCO_3_^−^ has been reached. This conclusion is further supported by experiments using the pan-carbonic anhydrase inhibitor acetazolamide. Under our buffered assay conditions 500 μM acetazolamide did not affect basal or rotenone-inhibited R-Dye generation in THP-1 monocytes (Supplemental Fig. 3E). Taken together, characterisation of the functionality of this sensor has provided unanticipated fundamental and previously uncharacterised insights into cellular CO_2_ production.

In hypercapnia pCO_2_ levels exceed 45 mmHg while normocapnic CO_2_ levels are currently considered to be 35-45 mmHg [31]. In hypercapnia studies, elevated CO_2_ levels are often simulated by exposing cells to 5% CO_2_ (35-45 mmHg, normocapnia) or 10% CO_2_ (>45 mmHg, a degree of hypercapnia detected in COPD and within the tumour microenvironment). Here we demonstrate the potential for CarboSenR2 to effectively differentiate between environments within this important range of CO_2_ concentrations spanning the transition between physiological and pathophysiological levels (Fig. 2A–D). This highlights CarboSenR2's potential for investigating the CO_2_ microenvironment within healthy tissues as well as solid tumours, where the pCO_2_ levels have been recorded to be as high as 84 mmHg [32].

The cationic nature of CarboSenR2, combined with its lipophilic properties due to aromatic rings, suggests it would be attracted to regions with a net negative charge, particularly the mitochondria. Interestingly, the green fluorescence of CarboSenR2 (Fig. 3, Fig. 4) reveals that the sensor is relatively uniformly distributed throughout the cytoplasm, it does not selectively accumulate in the mitochondria and is also clearly visible in the nucleus. Furthermore, the fluorescence of the R-Dye (indicative of CO_2_ presence) appears preferentially clustered around perinuclear regions, suggesting the existence of potential CO_2_-rich gradients within the cell (Fig. 3C). We hypothesised that these R-Dye ‘hotspots’ are mitochondrially associated (given that the mitochondria are the central hub of cellular CO_2_ production during aerobic metabolism) and tested this hypothesis using live cell fluorescent imaging. We observed that R-Dye fluorescence preferentially localised to regions abundant in mitochondria, with significantly less fluorescence localising within lysosomes (another organelle with peri-nuclear localisation) (Fig. 4). Notably, 91.75% (50X magnification) and 91.5% (100X magnification) of the R-Dye fluorescence signal overlapped with mitochondrial regions, while significantly less coincided with lysosomal regions (45.75% at 50X, 41% at 100X). Mitochondria are visually long and ‘string-like’ as evidenced by the Biotracker 405 staining (Fig. 4A and B). While not all R-Dye staining matches this pattern, we clearly observe ‘string-like’ R-Dye fluorescent staining in (Fig. 4B and C) which overlaps with the mitochondrial Biotracker 405 staining pattern. Together, this suggests that in living cells R-Dye fluorescence is emanating from mitochondria and mitochondria-associated cellular domains driven by a concentration gradient. This in turn leads to the generation of R-Dye ‘hot-spots’, demonstrating compartmentalised CO_2_ production by mitochondria. This concept is consistent with the seminal work of Balboni and Lehninger on rat liver mitochondria, that proposed that at pH 7.2, the major species exiting respiring mitochondria is dissolved CO_2_, rather than HCO_3_^−^ or H_2_CO_3_ [33].

In the physiological literature, the existence of intracellular CO_2_ gradients remain largely unexplored in mammalian systems, despite their presence in mesophylls of plants [34] whereby CO_2_ concentrating mechanisms are present [35]. Additionally, cellular oxygen gradients have been documented in mammalian cells using microscopy approaches [[36], [37], [38]]. It is proposed that under low oxygen conditions, pharmacological suppression of mitochondrial respiration with nitric oxide (NO) redirects oxygen toward non-respiratory, oxygen-dependent targets like prolyl hydroxylases to facilitate hypoxia inducible factor degradation, thereby supporting the concept of cellular oxygen gradients/oxygen re-distribution [39]. Altered oxygen gradients are linked to different degrees of aerobic respiration, which would logically lead to local changes in CO_2_ production. Thus, we provide evidence of R-Dye associated ‘hot-spots’ *in cellula* that reflect differences in mitochondrial activity – a theory which was previously proposed based on findings from isolated rat liver mitochondria using a gas permeable CO_2_-sensitive electrode [2,33].

As discussed, the enzymes responsible for CO_2_ production are primarily localised within the TCA cycle in the mitochondria. During the breakdown of isocitrate by IDH, alpha-ketoglutarate (α-KG) and CO_2_ are produced. Given R-Dye fluorescence has been shown to preferentially localise to mitochondria and peri-mitochondrial areas (Fig. 4), mitochondrial-targeted metabolic inhibitors were applied to investigate CO_2_ production, and its relationship with mitochondrial function. Interestingly, treatment of THP-1 monocytes with rotenone and oligomycin significantly decreased R-Dye fluorescence, leading us to hypothesise that endogenous CO_2_ production can be affected by interfering with oxidative phosphorylation (Fig. 5). Rotenone treatment significantly impacts active mitochondria, increases ROS production, and decreases ATP production [40]. Consequently, rotenone-induced suppression of mitochondrial activity leads to dysregulation of the TCA cycle primarily due to negative feedback mechanisms induced from NADH accumulation [41], likely impacting CO_2_ production. Oligomycin functions by inhibiting ETC-dependent ATP synthesis, resulting in mitochondrial dysfunction [42]. This dysfunction leads to the loss of ATP-dependent allosteric regulation, diminished ATP availability, and disruption of the ETC, further compromising mitochondrial function and aerobic metabolism [43]. As a consequence, the cell is forced to rely on alternative metabolic pathways, attempting to adapt to the energy deficit. In summary, treatments with rotenone, and oligomycin significantly decreased R-Dye fluorescence activity, suggesting a clear link between disruption of the mitochondrial electron transport chain and changes in intracellular CO_2_ production – findings which align with previous observations in isolated rat liver mitochondria, where a complex III inhibitor of the ETC (antimycin A) significantly impaired mitochondrial CO_2_ production, as measured by a CO_2_-sensitive electrode. [33]. While pharmacological inhibition of R-Dye generation has yielded intriguing results, we next sought to modulate mitochondrial function using more subtle/indirect approaches including treatment with 2-deoxy d-glucose and malonate. 2-deoxy-d-glucose (2-DG), is a synthetic glucose analogue that competitively inhibits glucose utilisation. Unlike glucose, 2-DG cannot undergo isomerisation by phosphoglucose isomerase due to the absence of a hydroxyl group at the second carbon, thereby blocking the initial steps of glucose metabolism and subsequently hindering both glycolysis and downstream glucose utilisation for aerobic metabolism [44]. We hypothesised that interference with glycolytic flux would decrease R-Dye fluorescence, indicative of suppressed CO_2_ production downstream in the TCA cycle. THP-1 monocytes treated with 6.125 mM glucose and 2-DG (5 mM) exhibited significantly diminished R-Dye fluorescence and cellular proliferation indicating that 2-DG caused substantial downstream effects on CO_2_ production. Disruption of TCA flux e.g. through malonate supplementation (8 mM) inhibits succinate dehydrogenase (SDH), decreased cellular proliferation and inhibited cellular R-Dye production. Importantly, the analysis of R-Dye fluorescence was only performed on live and viable cells supporting the concept that malonate supplementation inhibits cellular CO_2_ production. Thus, we provide evidence of a relationship between CO_2_ production and cell metabolism, suggesting a novel potential method for monitoring cellular function/proliferation through CO_2_ production rates.

Since the CarboSenR2 turn-on response was significantly impacted by multiple interventions that negatively affect cell metabolism, we next designed an experiment hypothesised to enhance aerobic metabolism, boost TCA cycle flux and evidentially, increase CO_2_ production. Exercise is both a mechanical and metabolic stressor. Aerobic exercise at moderate intensity is a well-established intervention known to drive O_2_ consumption, cellular metabolism, and mitochondrial biogenesis as well as CO_2_ production [45]. Devices such as Electrical Pulse Stimulators (EPS) mimic neuromuscular junction and synapse activity, promoting muscle contraction [46]. Thus, EPS is a known exercise mimetic for *in cellula* studies using C2C12 skeletal muscle models [45]. We observed an increase in R-Dye in mature myotubes that physically contract in response to EPS stimulation (Fig. 6A and B, Supplemental Fig. 9). To our knowledge, this is the first successful attempt to directly monitor metabolically produced molecular CO_2_ dynamics within exercising cells rather than relying on ΔpH, NaHCO_3_^−^, pCO_2_ or VCO_2_ measurements. Myoblasts exposed to EPS also increased R-Dye fluorescence after 1 h of stimulation. Interestingly, this EPS protocol did not significantly affect ΔΨm suggesting that changes in R-Dye fluorescence activation may be a more sensitive index of mitochondrial activity than ΔΨm as measured by Biotracker Blue. Thus, R-Dye fluorescence is increased in EPS-treated C2C12 skeletal muscle cells. This finding speaks to the sensitivity of CarboSenR2 in biological systems and also the potential utility of this sensor to monitor skeletal muscle metabolism *in cellula* in a manner that has not hitherto been possible.

Given the utility of CarboSenR2 to detect physiological levels of CO_2_ in monocytes and myoblasts we asked whether it could distinguish between immune cells derived from a common monocyte background that had been differentiated into mature macrophages of distinct metabolic profiles using distinct cytokine polarising protocols. Macrophages demonstrate metabolic plasticity depending on their polarisation state [26] – PMA-induced Mφ macrophages can be subsequently polarised with LPS and IFN-γ or IL-4 to induce pro-inflammatory (M1-like) and alternative/anti-inflammatory (M2-like) phenotypes, respectively. Classical (M1-like) macrophages predominantly rely on anaerobic metabolism, which does not generate CO_2_ due to its independence from molecular oxygen. In contrast, alternatively activated (M2-like) macrophages adopt a more aerobic phenotype, theoretically leading to an increased rate of CO_2_ production. Consistent with the current understanding of macrophage metabolism, we observed a significant decrease in R-Dye fluorescence, in M1-like macrophages, compared to both Mφ and IL-4 polarised, M2-like macrophages (Fig. 7C) suggesting lower endogenous CO_2_. Based on the understanding that Mφ exhibit greater oxidative and aerobic metabolic capacities compared to M1-like macrophages [47], a more complex model comprising both Mφ and M1-like THP-1 macrophages was developed to investigate whether heterogeneous cell populations containing subpopulations with distinct metabolic phenotypes could be reliably identified and separated based on differences in their endogenous CO_2_ concentrations. We observed a significant decrease in R-Dye fluorescence in M1-like macrophages when compared to Mφ macrophages, which is consistent with our previous findings (Fig. 7D). These data demonstrate that these cell phenotypes exhibit distinct metabolic characteristics, as evidenced by the analysis of CO_2_ production and align with previous reports highlighting the metabolic divergence between macrophage subtypes [26]. CarboSenR2's ability to differentiate between cell types based on metabolic activity underscores its potential for investigating tumour-like environments and quantifying cell-specific contributions to the development of hypercapnic microenvironments, which could serve as a distinguishing factor between malignant and non-malignant cells. Given that CO_2_ production would indeed be proportional to TCA cycle turnover rates (in a unidirectional manner), this experiment highlights the potential for using CO_2_ profiling as a means of investigating the metabolic profile of a specific cell type, especially when done in combination with another cell defining/distinguishing marker.

Finally, we focused our attention again on mitochondrial function and tested the sensitivity of CarboSenR2 to changes in mitochondrial function alongside two well established, standard methods of assessing mitochondrial mass and mitochondrial membrane potential. To do this we used IMT-1 to specifically inhibit mtDNA-dependent transcription [48]. This disrupts mitochondrial function [48], cellular activity [48], and ultimately impairs cell proliferation (Fig. 8B and D). Interestingly, IMT-1 did not affect cellular viability over chronic treatment periods, except at the highest dose tested, 1.6 μM (Fig. 8C) as measured by Trypan Blue staining. Interestingly, changes in R-Dye fluorescence were observed at much lower doses compared to the levels at which mitochondrial mass (as measured by NAO fluorescence) and mitochondrial membrane potential (as measured by Biotracker Blue) were affected (Fig. 8E). Thus, given that CO_2_ production is a key event in aerobic metabolism, its measurement with CarboSenR2 may serve as a sensitive readout of mitochondrial dysfunction even before significant changes in membrane potential or cardiolipin levels are observed. This suggests that changes in CO_2_ production could serve as an earlier and potentially more reliable indicator of mitochondrial integrity, providing a window for early-stage detection of cellular distress. This could widen the window for intervention to improve therapeutic or treatment opportunities before more significant or lasting effects develop. Thus, CarboSenR2 represents a tool that has been sorely lacking in the field of CO_2_ and redox biology to date.

## 5 Limitations

Our data indicate that CarboSenR2 is capable of detecting CO_2_ levels *in cellula*, giving new insights into cellular metabolism. Despite this, there are several limitations that need to be considered when using this sensor. (i) CarboSenR2 is pH sensitive, thus changes in green fluorescence intensity can be due to probe abundance or local pH. For this reason CarboSenR2 imaging is best limited to the confirmation of cell loading (Supplemental Fig. 1A) (ii) the conversion of CarboSenR2 to R-Dye is pH sensitive (Supplemental Fig. 1C) – this needs to be considered when working with biological systems with a pH of </ = 6. (iii) CarboSenR2 can cause cytotoxicity at concentrations >/ = 5 μM in certain cells (Supplemental Fig. 3C and D). Therefore low dose loading is recommended, and users should optimise their loading for their specific cell type/biological system. Direct quantitative differences in R-Dye are best determined between experiments performed in parallel with all loading and washing conditions standardised. (iv) CarbsenR2 is subject to hydrolysis, for this reason experiments should be short duration (up to 2-3 h) (Fig. 2B and C) and users should rely on the R-Dye readout rather than a ratio of CarboSenR2:R-Dye (v) CarboSenR2-derived R-Dye formation rate is temperature sensitive (Supplemental Fig. 4). If experiments are performed at low temperatures e.g. on ice the rate of conversion will also be influenced by temperature (vi) CarboSenR2 is not currently formulated to target specific intracellular organelles, future work may allow for organelle specific measurements of CO_2_/R-Dye (vii) while it is attractive to speculate that R-Dye ‘hotspots’ are direct evidence of CO_2_ microdomains, this is not conclusively demonstrated here as CarboSenR2 is not 100% freely diffusible *in cellula* and likely interacts with intracellular biomolecules/proteins. Furthermore, we cannot fully exclude the possibility that R-Dye may accumulate in mitochondria having being produced elsewhere in the cell, and that R-Dye accumulation in the mitochondria can potentially influence mitochondrial membrane potential (thus, we suggest caution interpreting data involving mitochondrial uncoupling agents). Future generation of CO_2_ sensors could address some of these limitations (viii) Changes in R-Dye fluorescence were of a higher magnitude with inhibition of mitochondrial function, compared to stimulation of mitochondrial function. This may reflect sensitivity of the probe and/or the fact that it is easier to markedly inhibit mitochondrial function as opposed to stimulate mitochondrial function within the timeframe of our experiments.(ix) Quantitative analysis using CarboSenR2 is best achieved using flow cytometry (as opposed to microscopy and HPLC which are more useful for qualitative analysis).

## 6 Conclusion

CO_2_ is a ubiquitous physiological gas, and its endogenous production and roles are unresearched at least in part due to a lack of reliable tools to directly study the gas in living systems. CarboSenR2 is a new selective CO_2_ fluorescent sensor that has been described to image exogenous CO_2_ *in vitro* but requires much further evaluation *in cellula*. Here, we demonstrate its utility as a CO_2_ sensor in multiple cell systems using flow cytometric and microscopy based approaches. These data demonstrate reliable CO_2_ sensitivity of the sensor within the physiological to pathophysiological range (5-10% CO_2_) and provide evidence of mitochondrial associated R-Dye ‘hot-spots’ within cells. CarboSenR2 fluorescence activation is closely linked to mitochondrial function as suppressing mitochondrial activity with pharmacological or metabolic modulators decreases the R-Dye fluorescence. Stimulating aerobic metabolism by applying exercise-like stimuli with EPS, concordantly increased fluorescence response of CarboSenR2. Intriguingly, R-Dye fluorescence mapped to crucial biological processes including cell proliferation rate and macrophage metabolic phenotype. Thus, CarboSenR2 may be of broad utility as a sensitive and early-stage marker of cellular and mitochondrial function/dysfunction. We believe that CarboSenR2 -based assays can serve as a complementary approach to the study of cellular metabolism, and mitochondrial function/dysfunction alongside traditional respirometry based approaches. This is important because of CO_2_'s reactivity with protein targets (carbamylation) [9,12,49], peroxides (peroxymonocarbonate HCO_4_^−^) and peroxinitrites (nitrosoperoxocarboxylate (ONOOCO_2_^−^) [2]. Furthermore, CO_2_ can be formed in pathways independent of respiration and mitochondria e.g. via cytoplasmic activity of prolyl hydroxylases (PHDs) to regulate the stability of Hypoxia-Inducible Factor Alpha- HIFα [50] or independent of oxygen entirely e.g. via the activity of histidine decarboxylases [51]. Thus, measuring cellular CO_2_ using probes such as CarboSenR2 provides an orthogonal and alternative readout of cellular metabolism that may reveal new biological insights that are not fully captured by existing traditional respirometry based assays.

## CRediT authorship contribution statement

**Ben Reddan:** Conceptualization, Formal analysis, Investigation, Methodology, Project administration, Validation, Visualization, Writing – original draft, Writing – review & editing. **Rawan Shahen:** Data curation, Formal analysis, Investigation, Methodology, Writing – original draft, Writing – review & editing. **Rafael Radi:** Conceptualization, Methodology, Writing – original draft, Writing – review & editing. **Mia McCalmont:** Investigation, Methodology, Writing – review & editing. **Ori Green:** Conceptualization, Formal analysis, Investigation, Methodology, Resources, Supervision, Validation, Writing – original draft, Writing – review & editing. **Eoin P. Cummins:** Conceptualization, Formal analysis, Funding acquisition, Investigation, Methodology, Project administration, Supervision, Writing – original draft, Writing – review & editing.

## Declaration of competing interest

O.G. is an inventor on patent application US18723792 related to the CO_2_ sensing technology CarboSen.

The other authors declare that they have no conflict of interest with the contents of this article.

## Data Availability

Data will be made available on request.
