# Supplementary material for: Activity-based CO2 sensing using CarboSenR2 provides new insights into cellular metabolism
Source: Redox Biol. 2026 Feb 4;91:104067. doi: 10.1016/j.redox.2026.104067 (PMC12907856; doi:10.1016/j.redox.2026.104067)
Supplement: Multimedia component 1 [file mmc1.docx]

**Supplemental Figures**

**
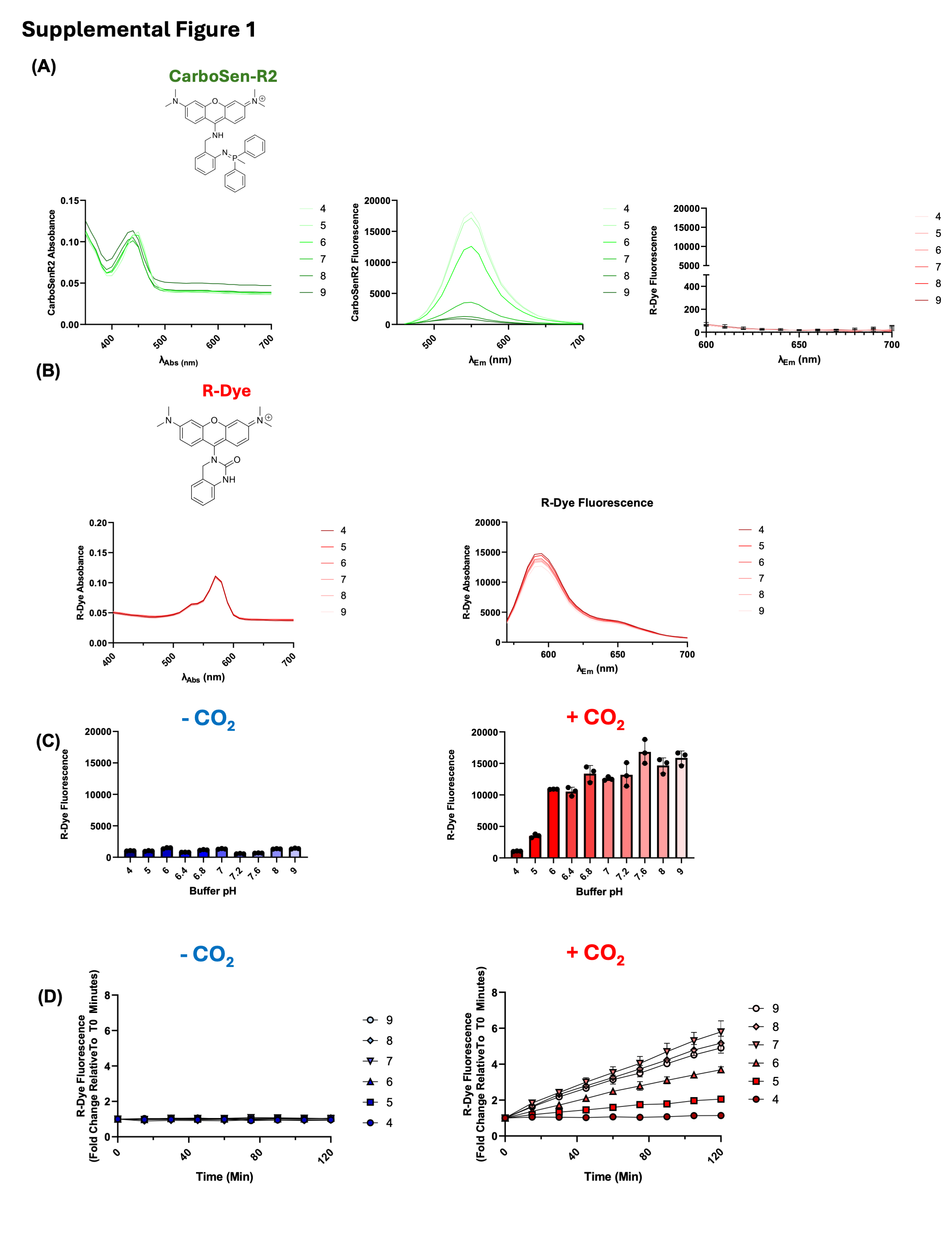
**

**
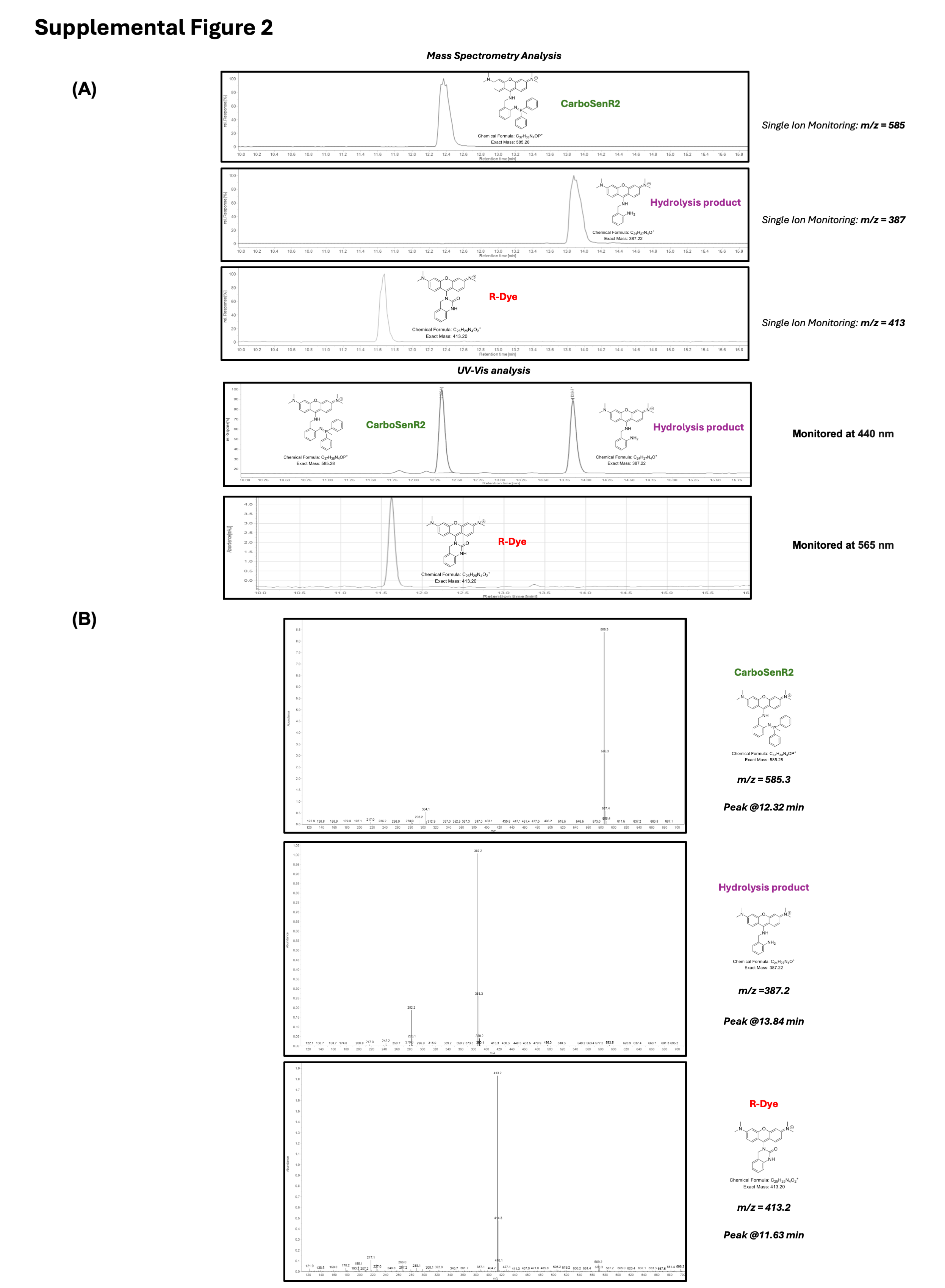
**

**
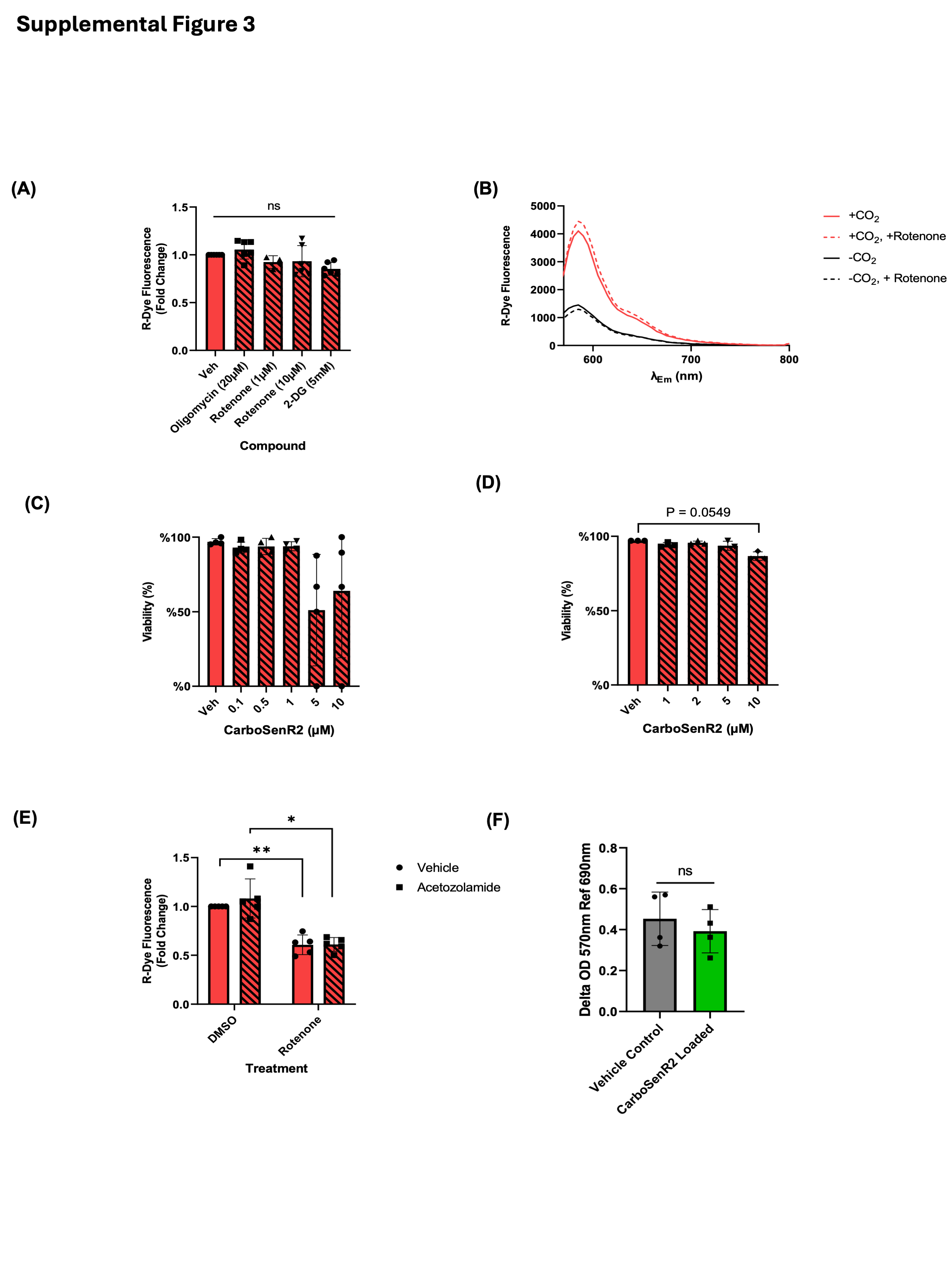
**

**
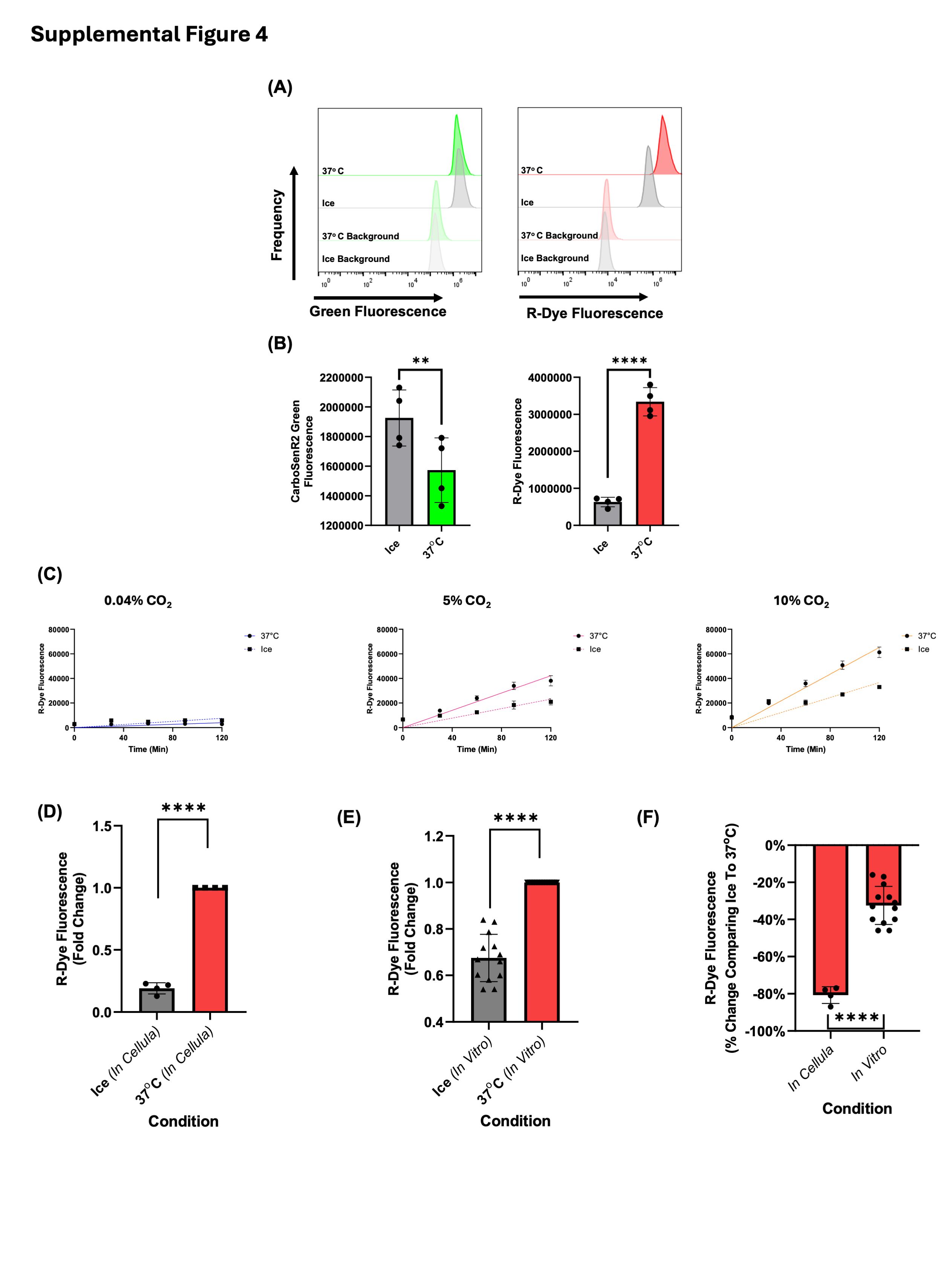
**

**
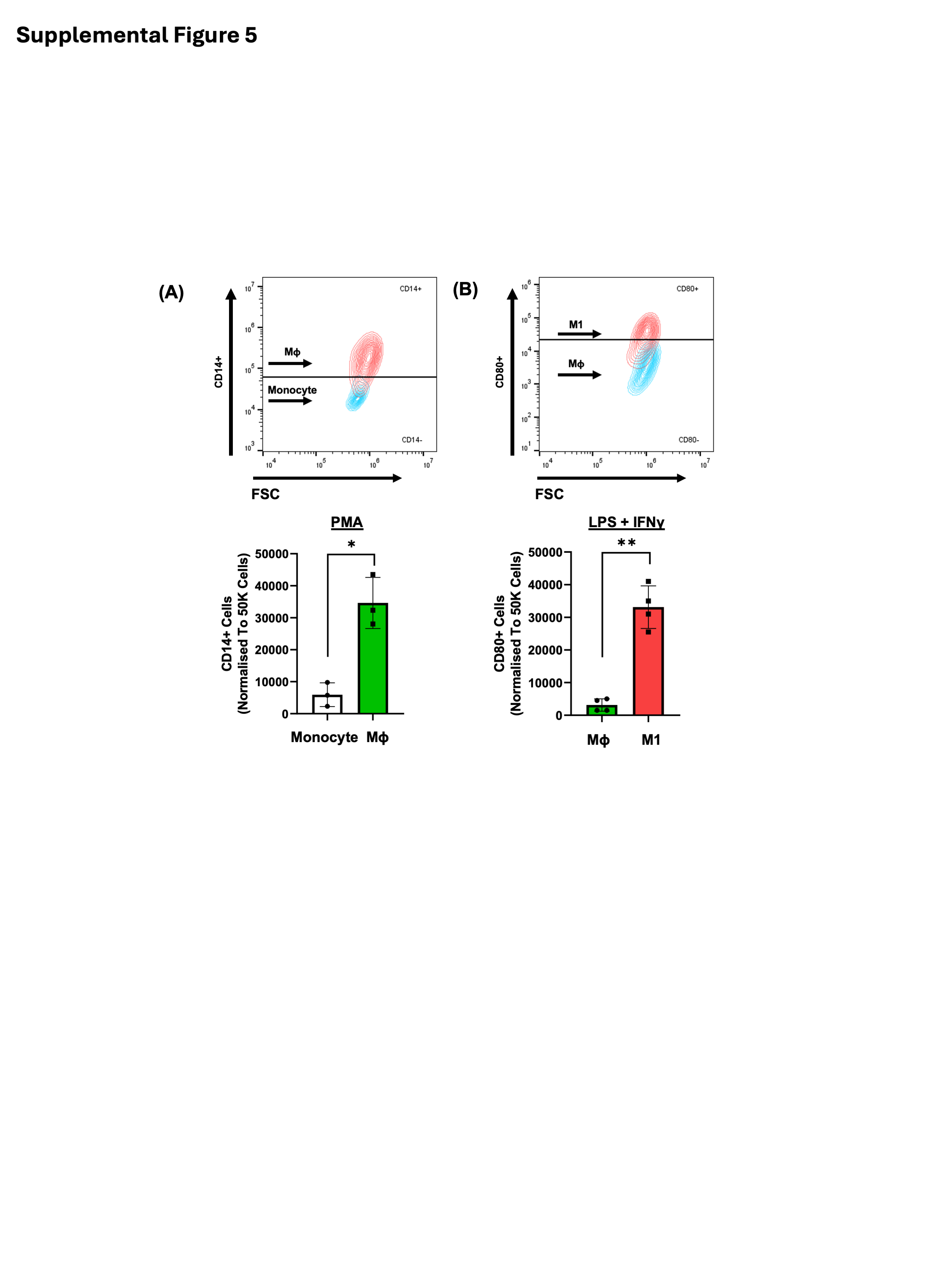
**

**
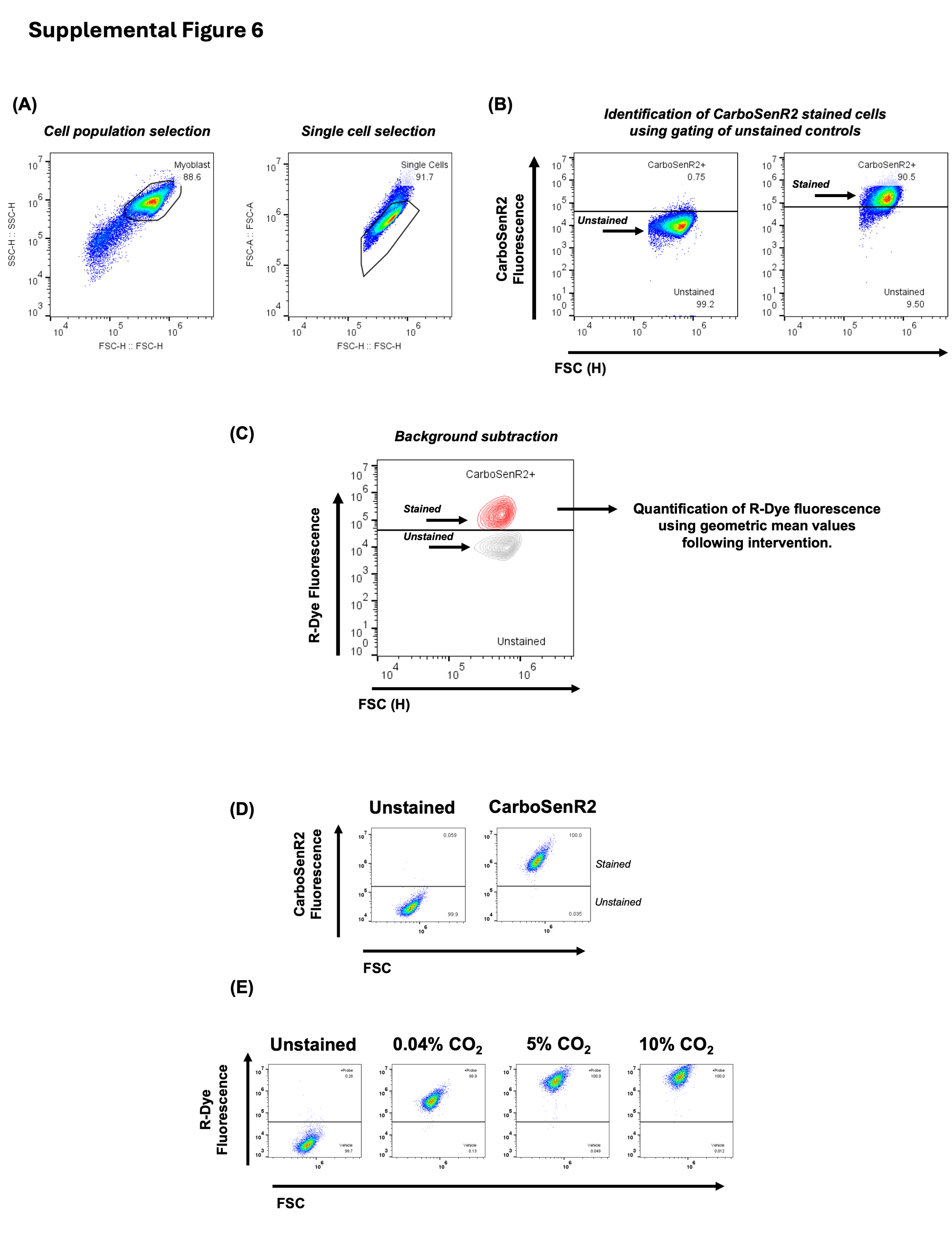
**

**
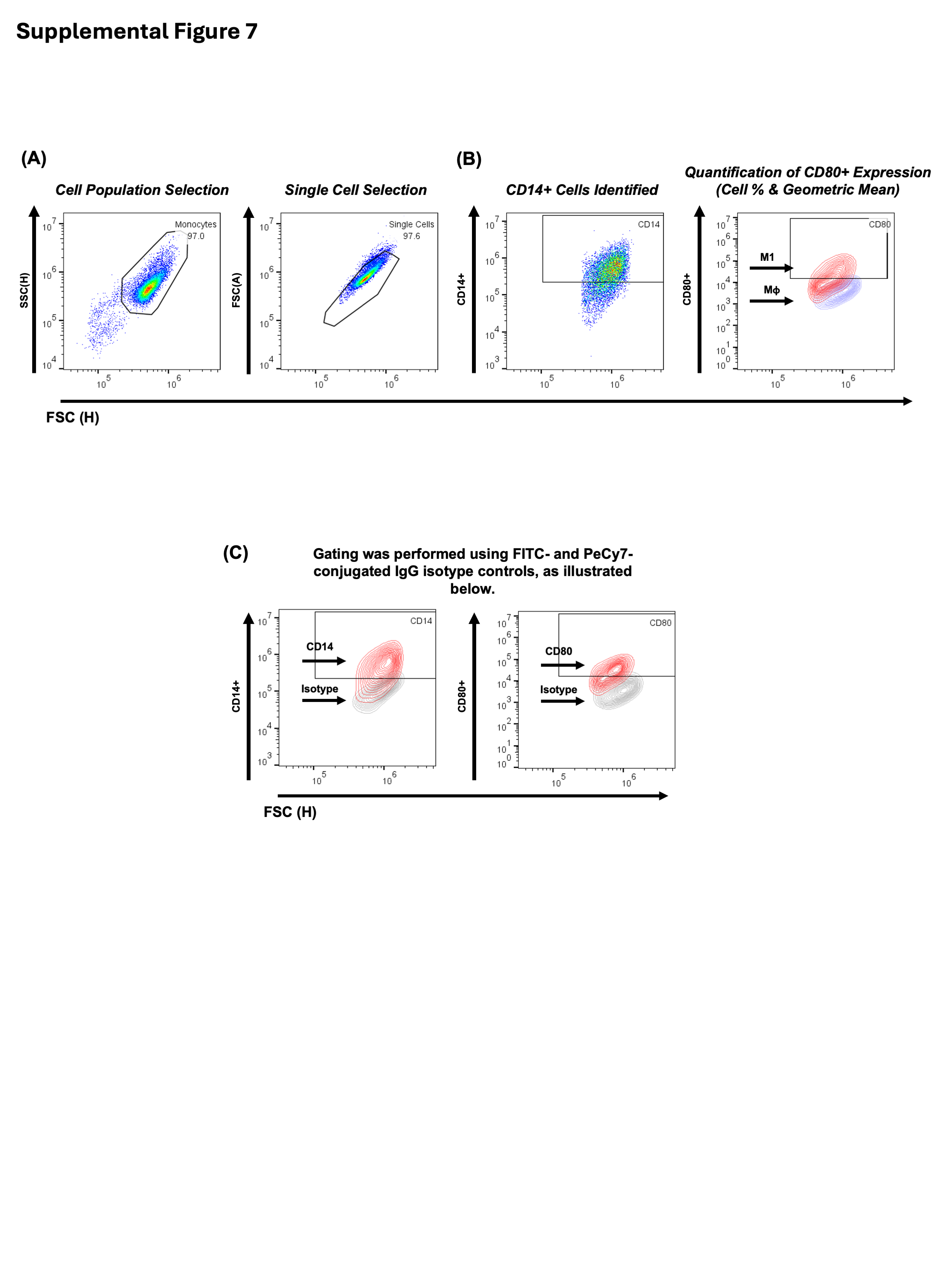
**

**
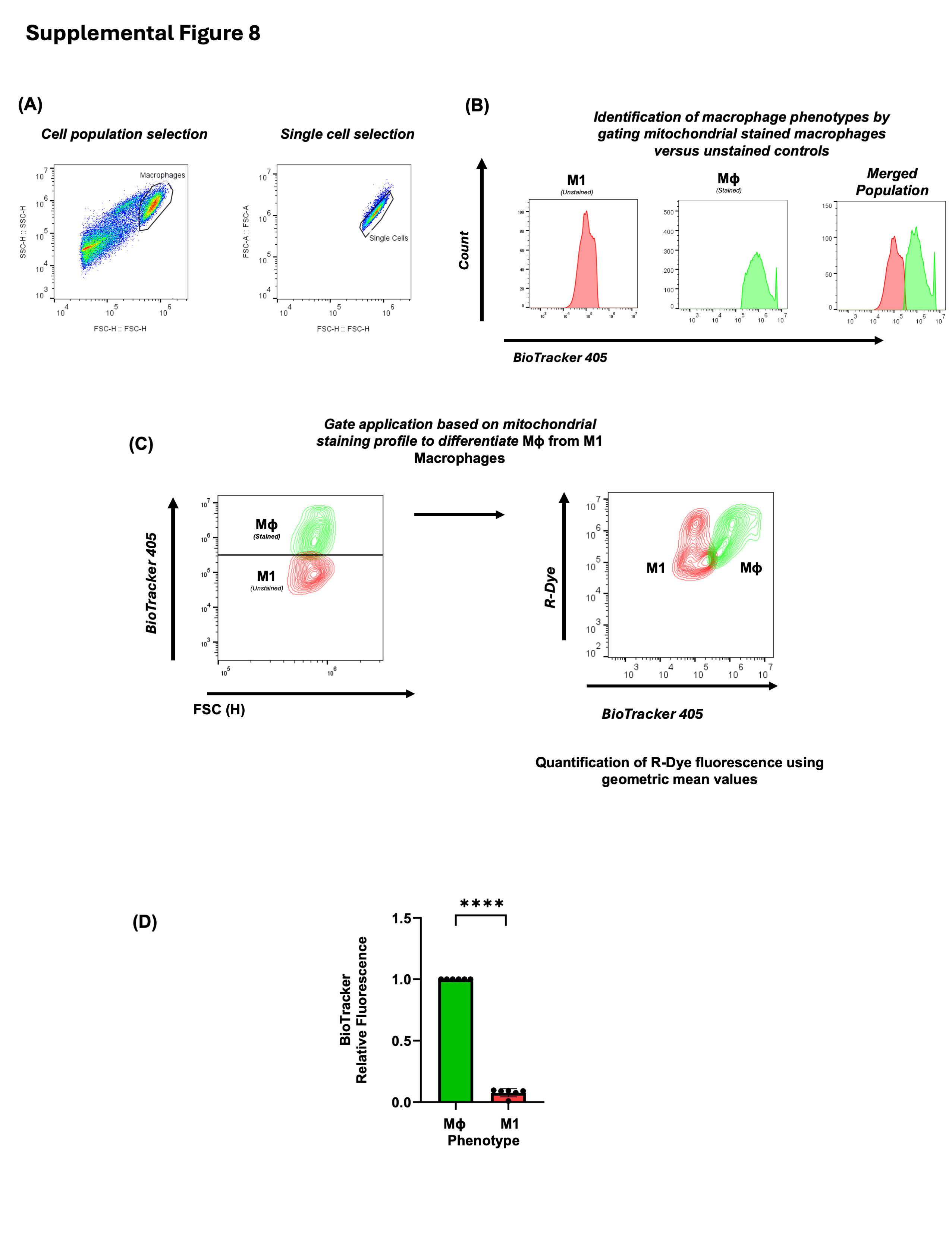
**

**
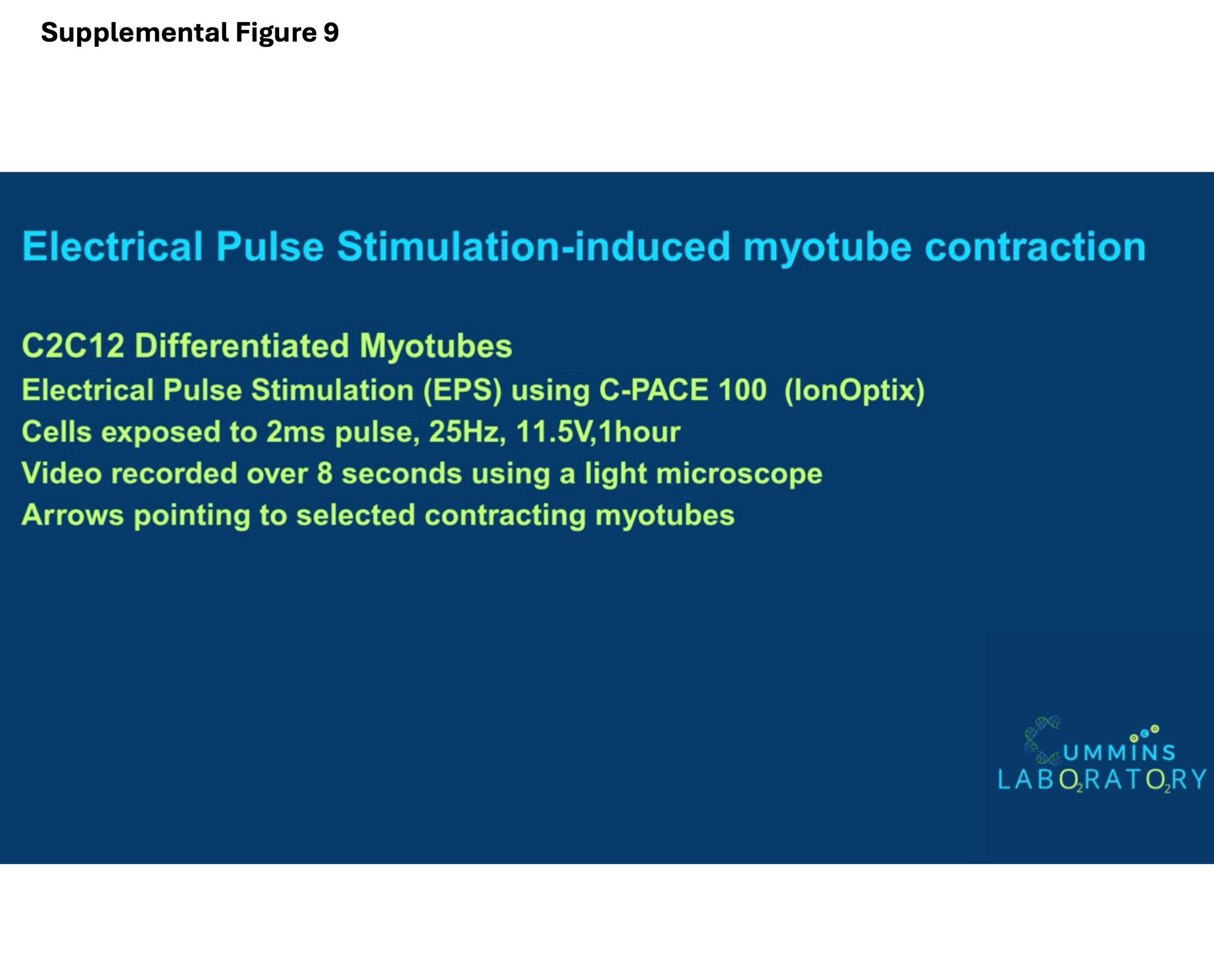
**

[**Link to video**](https://www.canva.com/design/DAG4BKpiJ1c/6okaYji8ExnhiUCBkaD7uw/watch?utm_content=DAG4BKpiJ1c&utm_campaign=designshare&utm_medium=link2&utm_source=uniquelinks&utlId=h0af128c748)

**
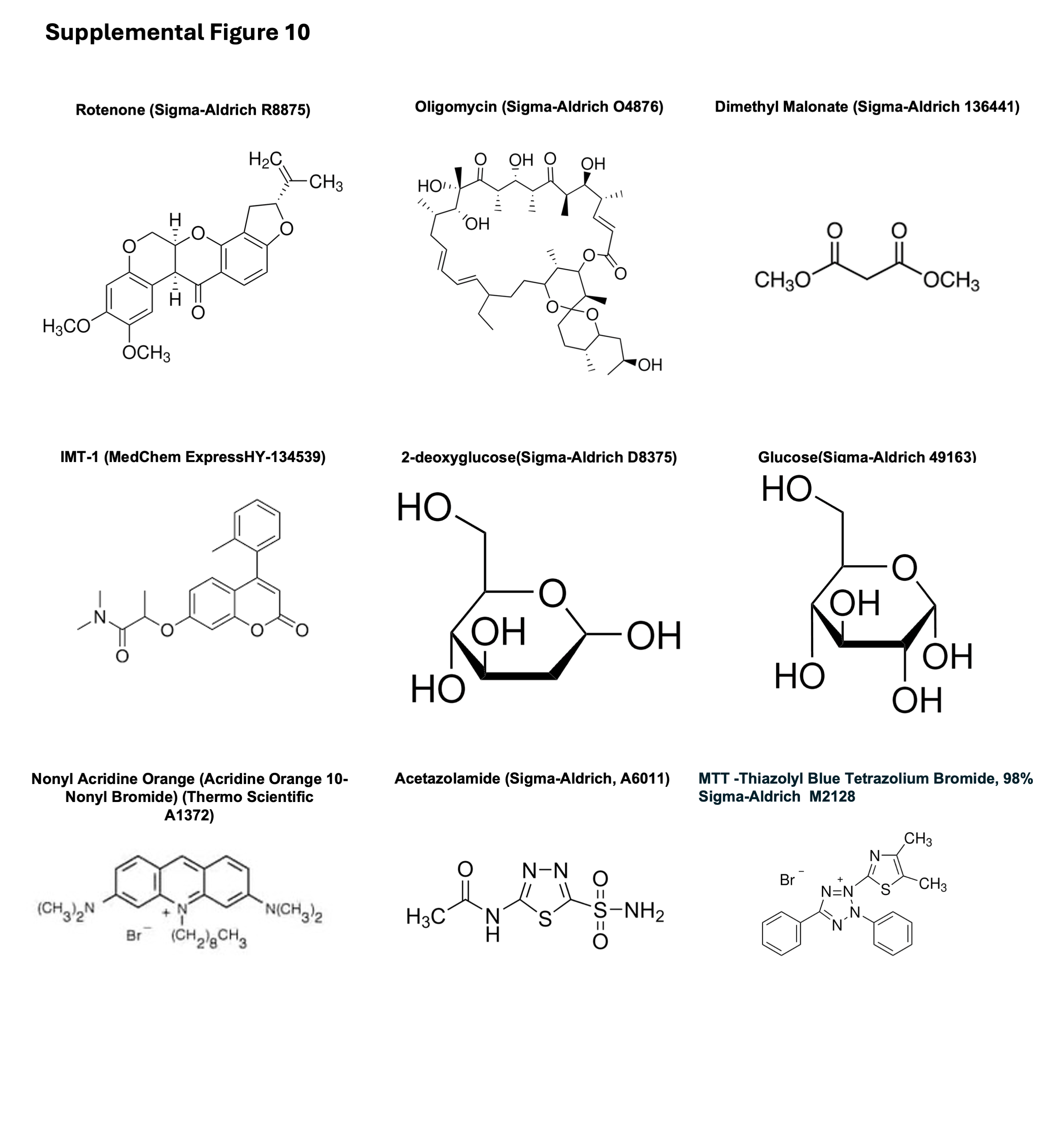
**

**
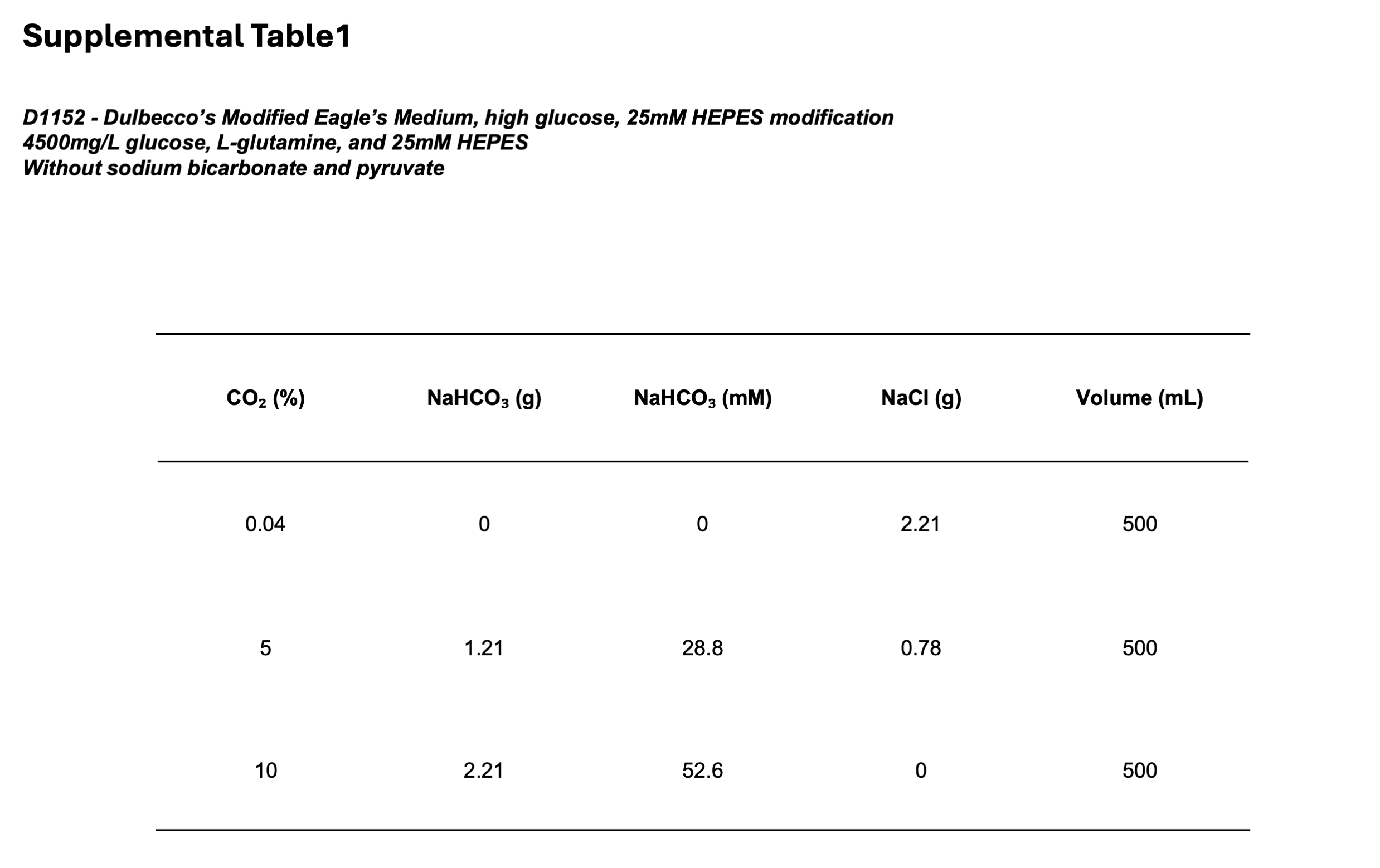
**
